# LRRK2 Gly2019Ser Mutation Promotes ER Stress via Interacting with THBS1/TGF‐β1 in Parkinson's Disease

**DOI:** 10.1002/advs.202303711

**Published:** 2023-09-06

**Authors:** Longping Yao, Fengfei Lu, Sumeyye Koc, Zijian Zheng, Baoyan Wang, Shizhong Zhang, Thomas Skutella, Guohui Lu

**Affiliations:** ^1^ Department of Neurosurgery First Affiliated Hospital of Nanchang University Nanchang 330209 P. R. China; ^2^ Department of Neurosurgery Zhujiang Hospital Southern Medical University Guangzhou 510282 P. R. China; ^3^ Institute for Anatomy and Cell Biology Medical Faculty Heidelberg University 69120 Heidelberg Germany; ^4^ Department of Neuroscience Institute of Health Sciences Ondokuz Mayıs University Samsun 55139 Turkey

**Keywords:** endoplasmic reticulum (ER) stress, LRRK2 G2019S, Parkinson's disease, TGF‐β1, THBS1

## Abstract

The gene mutations of LRRK2, which encodes leucine‐rich repeat kinase 2 (LRRK2), are associated with one of the most prevalent monogenic forms of Parkinson's disease (PD). However, the potential effectors of the Gly2019Ser (G2019S) mutation remain unknown. In this study, the authors investigate the effects of LRRK2 G2019S on endoplasmic reticulum (ER) stress in induced pluripotent stem cell (iPSC)‐induced dopamine neurons and explore potential therapeutic targets in mice model. These findings demonstrate that LRRK2 G2019S significantly promotes ER stress in neurons and mice. Interestingly, inhibiting LRRK2 activity can ameliorate ER stress induced by the mutation. Moreover, LRRK2 mutation can induce ER stress by directly interacting with thrombospondin‐1/transforming growth factor beta1 (THBS1/TGF‐β1). Inhibition of LRRK2 kinase activity can effectively suppress ER stress and the expression of THBS1/TGF‐β1. Knocking down THBS1 can rescue ER stress by interacting with TGF‐β1 and behavior burden caused by the LRRK2 mutation, while suppression of TGF‐β1 has a similar effect. Overall, it is demonstrated that the LRRK2 mutation promotes ER stress by directly interacting with THBS1/TGF‐β1, leading to neural death in PD. These findings provide valuable insights into the pathogenesis of PD, highlighting potential diagnostic markers and therapeutic targets.

## Graphical abstract

Leucine‐rich repeat kinase‐2 (LRRK2) Gly2019Ser promotes endoplasmic reticulum (ER) stress and neural death in Parkinson's disease (PD) by interacting with thrombospondin‐1/transforming growth factor beta1 (THBS1/TGF‐β1) in dopamine neurons and mice. Inhibiting LRRK2 activity reduces ER stress. Targeting THBS1 or TGF‐β1 rescues ER stress and behavioral burden caused by the mutation, offering potential therapeutic targets for PD.

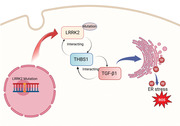

## 1 Introduction

The aberrant deposits of α‐synuclein aggregates in the brain and the degeneration of dopamine (DA) neurons are clinical manifestations of Parkinson's disease (PD) in the substantia nigra pars compacta (SNpc).^[^ ^1^ ^]^ The hallmark motor manifestations of PD include bradykinesia (slowness of movement), rigidity, resting tremors, and postural instability, all of which significantly impact the patient's ability to carry out daily activities.^[^ ^2^ ^]^ The most prevalent pathogeny of PD with autosomal‐dominant hereditary is leucine‐rich repeat kinase 2 (LRRK2) missense mutations.^[^ ^3^ ^]^ This gene has been identified as a prominent contributor in patients with familial and sporadic PD.^[^ ^2^ ^]^ Because the long‐term danger of LRRK2 mutations is predicted to be 22%–32% in PD patients, the penetrance with LRRK2 mutations of PD is incomplete, implying important modifiers of LRRK2 illness.^[^ ^2^ ^]^ Patients with apparent sporadic PD and autosomal dominant PD patients, who are virtually indistinguishable from people suffering from idiopathic PD, are found to have the mutations of LRRK2, peculiarly the most prevalent variant Gly2019Ser (G2019S).^[^ ^3^ ^]^ The G2019S LRRK2 mutation is in most LRRK2‐PD patients, and it has been associated with neuropathological abnormalities comparable to those seen in idiopathic PD.^[^ ^4^ ^]^ Typical aggregates of synuclein are among these changes in the form of Lewy bodies and neurites, as well as neuron death in sensitive locations.^[^ ^5^ ^]^

LRRK2, a 286 kDa protein, has repetitive motifs on the N‐terminal half, and guanosine triphosphatases  and kinase domains are around with interaction domains of protein to the protein on the C‐terminal half.^[^ ^6^ ^]^ The two catalytic domains contain the most harmful mutations, but they all cause the kinase to become hyperactive in cells.^[^ ^7^ ^]^ Reduced LRRK2 activity or expression is neuroprotective in preclinical research. The inhibitors of LRRK2 and antisense oligonucleotides in small molecules have been invented and are currently deemed safe for therapeutic usage.^[^ ^4a^ ^]^ However, it is yet unknown how the activity of LRRK2 kinase is maintained at the cellular and molecular levels and how the abnormal activity of LRRK2 kinase correlates to PD.

Endoplasmic reticulum (ER) stress is a cellular response to various stimuli, such as protein misfolding, that disrupts the normal function of the ER.^[^ ^8^ ^]^ ER stress triggers the unfolded protein response (UPR), a signaling pathway that attempts to restore ER homeostasis by reducing protein synthesis, increasing protein folding capacity, and degrading misfolded proteins.^[^ ^8^ ^]^ If the UPR fails to restore ER homeostasis, ER stress can activate cell death pathways and contribute to the pathogenesis of various diseases, including neurodegenerative disorders like PD.^[^ ^8^ ^]^ Previous studies have suggested that ER stress may play a role in the development of PD. For example, studies have shown that proteins associated with PD, such as α‐synuclein, can induce ER stress and activate the UPR in neuronal cells.^[^ ^9^ ^]^ The LRRK2 G2019S mutation is one of the most common genetic risk factors for PD, and its role in promoting ER stress in DA neurons has been suggested in several studies.^[^ ^10^ ^]^ In this study, we investigated the effects of the LRRK2 G2019S mutation on ER stress in induced pluripotent stem cell (iPSC)‐induced DA neurons and mice and identified potential therapeutic targets for PD.

This study applied Gene Expression Omnibus (GEO) datasets and validated the top gene targets affected by the LRRK2 mutation. We identified cluster of differentiation 44 (CD44), connective tissue growth factor (CTGF), thrombospondin‐1 (THBS1), vascular endothelial growth factor‐A (VEGFA), and secreted phosphoprotein‐1 (SPP1) as the potential genetic effectors responding to the mutation of LRRK2. We also investigated the effects of the LRRK2 G2019S mutation on ER stress in iPSC‐derived DA neurons and LRRK2 G2019S mice and explored the potential therapeutic targets for PD. Second, we sought to explore the relationship between LRRK2 G2019S and key molecules involved in ER stress regulation, particularly THBS1 and transforming growth factor beta‐1 (TGF‐β1). The identification of direct interactions between LRRK2 and these critical signaling molecules could shed light on novel therapeutic targets for PD. By elucidating the molecular crosstalk between LRRK2 and THBS1/TGF‐β1, we aimed to provide insights into the intricate signaling pathways that govern ER stress and neuron death in PD. Understanding these interactions could open up new avenues for developing targeted therapies to mitigate ER stress and its downstream effects, potentially halting or slowing disease progression in PD.

## 2 Results

### 2.1 LRRK2 G2019S Mutation Promotes ER Stress in iPSC‐Induced DA Neurons

**Figure 1:**
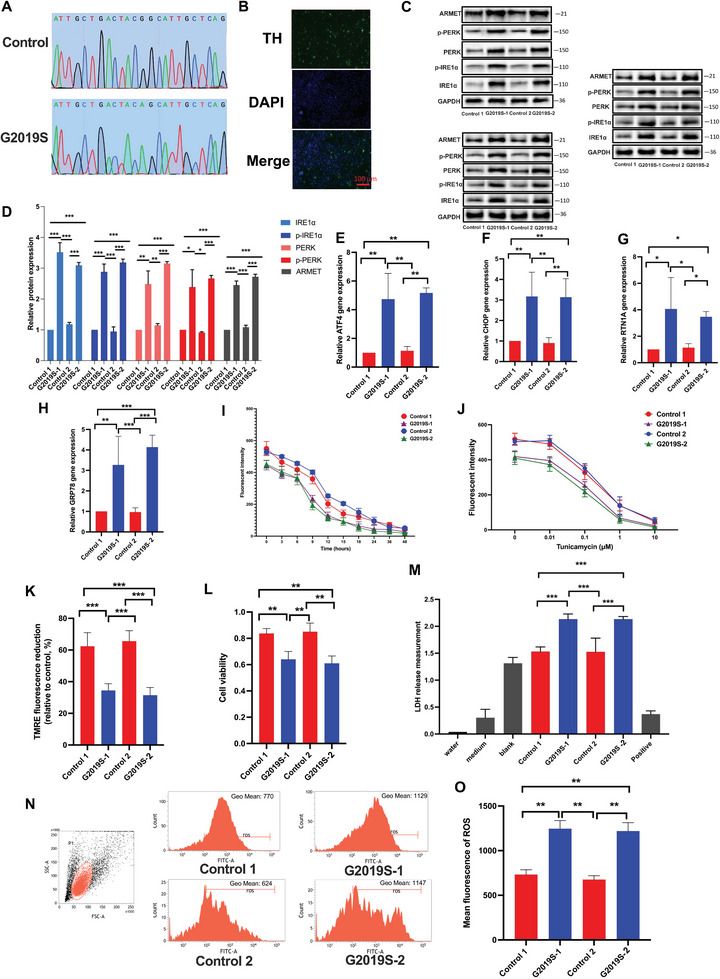
LRRK2 G2019S mutation promotes ER stress in iPSC‐induced DA neurons. A) Genomic deoxyribonucleic acid (DNA) sequencing reveals the presence of heterozygous G2019S mutation in two G2019S patient‐derived DA neurons. B) A confocal image confirmed the expression of TH supported by immunofluorescence. Green: anti‐TH (antibody labeling DA). Blue: DAPI. Scale bar, 100 µm. The protein expression of arginine‐rich, mutated in early‐stage tumors (ARMET), phospho‐protein kinase‐like ER kinase (p‐PERK), PERK, phospho‐inositol requiring enzyme 1 alpha (p‐IRE1α), and IRE1α were determined in triplicate by using C) western blot (WB) analysis and D) their relative expression was calculated. The intensity of the protein bands was normalized to glyceraldehyde‐3‐phosphate dehydrogenase (GAPDH). Reverse transcription‐quantitative real‐time polymerase chain reaction (RT‐qPCR) analysis of E) ATF4, F) CHOP, G) RTN1A, and H) GRP78 mRNA expression and normalized to the expression of GAPDH. I,J) The DA cells were subjected to pre‐treatment with Tunicamycin at a concentration of 1 µm for various time intervals (0, 3, 6, 9, 12, 15, 18, 24, and 48 h), or with Tunicamycin for 24 h at different concentrations (0, 0.01, 0.1, 1, and 10 µm). Fluorescent spectrophotometer traces were recorded, displaying the changes in Mag‐Fluo‐4 acetoxymethyl (AM) fluorescence intensity in triglyceride (TG)‐treated and CDN 1163 (CDN)‐treated DA cells in comparison to control cells. K) Mitochondrial membrane depolarization in WT and LRRK2 G2019S DA cells was assessed by measuring the fluorescence intensity of tetramethylrhodamine ethyl ester (TMRE) dye (250 nm). L) Cell viability was assessed using the cell counting kit‐8 (CCK‐8) assay. M) Cell death was determined by lactate dehydrogenase (LDH) assay. The level of ROS was studied using N) flow cytometry analysis, and O) relative intensity of ROS was calculated. Data were presented as means ± SD. The experiments were carried out three times (*n* = 3). One‐way analysis of variance (ANOVA) followed by Tukey's multiple comparison tests in (D, E, F, G, H, K, L, M, O). The difference in folds is statistically significant. ^*^ *P* < 0.05, ^**^ *P* < 0.01, ^***^ *P* < 0.001.

We enrolled two wild‐type (WT) healthy volunteers who had not been diagnosed with a central nervous system disease, and 2 PD patients who had the LRRK2 G2019S mutation verified (**Figure** **1**A). The WT fibroblast and PD patient fibroblasts with LRRK2 G2019S mutation were reprogrammed to iPSCs. Further, the iPSCs were differentiated into DA neurons. We validated the DA neurons' differentiation with tyrosine hydroxylase (TH) staining (Figure 1B). A previous study reported the mutation of LRRK2 resulted in cell toxicity and apoptosis in PD.^[^ ^11^ ^]^ Meanwhile, ER stress is a universal intracellular stress response caused by a range of situations that disrupt cellular homeostasis and leads to cell death.^[^ ^12^ ^]^ We further studied whether LRRK2 mutation could affect the ER stress in iPSC‐induced DA neurons. Specifically, compared with the LRRK2 wild group, the mutation of LRRK2 could significantly induce ER stress in the cells (Figure 1C,D). Simultaneously, we observed a significant upregulation in activating transcription factor 4 (ATF4), CCAAT/enhancer binding protein homologous protein (CHOP), reticulon 1A (RTN1A), and glucose‐regulated protein 78 (GRP78) messenger ribonucleic acid (mRNA) expression levels, suggesting a pronounced elevation in ER stress levels (Figure 1E–H).

Calcium ion (Ca^2+^) plays a vital role in the luminal biochemistry of the ER, particularly in relation to the calcium‐dependent chaperones responsible for folding newly synthesized proteins within this cellular compartment.^[^ ^13^ ^]^ Depletion of ER calcium levels hampers the proper folding capacity of these chaperones, leading to an accumulation of misfolded proteins and triggering ER stress.^[^ ^13^ ^]^ We employed tunicamycin to induce ER stress in DA neuron cells, followed by an examination of intracellular Ca^2+^ levels. The results indicate that varying stimulation times and concentrations of tunicamycin lead to a significant decrease in Ca^2+^ levels within DA neurons. Notably, we observed a more pronounced stimulation response to tunicamycin in the LRRK2 G2019S group when compared to the control group, suggesting an increased sensitivity to ER stress induction in the presence of the LRRK2 G2019S mutation (Figure 1I,J). ER stress‐induced Ca^2+^ release into cytosol causes the inner mitochondrial membrane depolarization, leading to mitochondrial reactive oxygen species (ROS) formation.^[^ ^14^ ^]^ We evaluated mitochondrial function by measuring alterations in the mitochondrial membrane potential. Our findings revealed a significant impairment in mitochondrial function observed in the LRRK2 G2019S group (Figure 1K).

What's more, if the UPR of ER stress is unable to successfully resolve cellular stress caused by protein misfolding or accumulation, it can trigger a proapoptotic program.^[^ ^13^ ^]^ Remarkably, our investigation demonstrated that the LRRK2 G2019S mutation exerts a notable influence on the promotion of apoptosis in DA neurons (Figure 1L,M; Figures S1A and S2B, Supporting Information). Meanwhile, we observed a significant increase in the release of ROS in cells carrying the LRRK2 G2019S mutation when compared to the control group (Figure 1N,O).

### 2.2 Inhibition of the Kinase of LRRK2 Suppresses ER Stress

**Figure 2:**
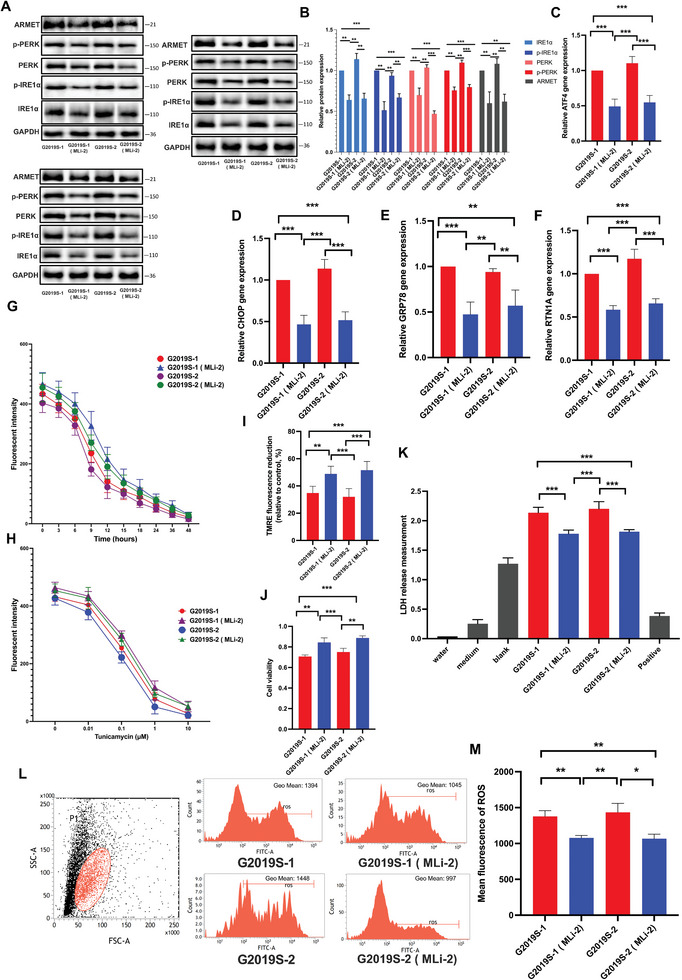
Inhibition the kinase of LRRK2 suppresses ER stress. The MLi‐2 compound was employed as a pharmacological inhibitor to target and inhibit the kinase activity of LRRK2. The protein levels of ARMET, p‐PERK, PERK, p‐IRE1α, and IRE1α proteins were analyzed in triplicate through A) WB analysis and B) their relative expression was calculated. The intensity of the protein bands was normalized to GAPDH. RT‐qPCR was performed to measure the levels of C) ATF4, D) CHOP, E) GRP78, and F) RTN1A. The resulting mRNA expression values were normalized to the expression of GAPDH. G,H) The DA cells were exposed to Tunicamycin at a concentration of 1 µm for varying time intervals ranging from 0 to 48 h. Additionally, another set of cells was treated with Tunicamycin for 24 h at different concentrations, including 0, 0.01, 0.1, 1, and 10 µm. Fluorescent spectrophotometer traces were recorded, displaying the changes in Mag‐Fluo‐4 AM fluorescence intensity in TG‐treated and CDN‐treated DA cells in comparison to control cells. I) Mitochondrial membrane depolarization in WT and LRRK2 G2019S DA cells was assessed by measuring the fluorescence intensity of TMRE dye at a concentration of 250 nm. J) Cell viability was assessed using the CCK‐8 assay. K) Cell death was determined by LDH assay. Flow cytometry analysis was utilized to study the level of L) ROS (L), and relative intensity of M) ROS was calculated. Data were presented as means ± SD. The experiments were carried out three times (*n* = 3). One‐way ANOVA followed by Tukey's multiple comparison tests in (B, C, D, E, F, I, J, K, M). The difference in folds is statistically significant. ^*^P < 0.05, ^**^P < 0.01, ^***^ *P* < 0.001.

The G2019S mutations result in an elevation of kinase activity in LRRK2.^[^ ^15^ ^]^ To investigate the potential involvement of kinase activity in modulating ER stress in LRRK2 mutant DA neurons, we administered a potent and well‐characterized LRRK2 kinase inhibitor, MLi‐2, to LRRK2 G2019S neurons.^[^ ^16^ ^]^ This experimental approach allowed us to assess the impact of kinase function on ER stress modulation in the context of LRRK2 G2019S mutation. The results demonstrated that the inhibition of LRRK2 kinase activity reduced the expression and phosphorylation of ER stress proteins (**Figure** **2**A,B). Likewise, the application of MLi‐2 significantly inhibited the mRNA expression of ATF4, CHOP, RTN1A, and GRP78, which are associated with ER stress (Figure 2C–F).

In the subsequent step, we pre‐treated DA neurons with MLi‐2 to reduce the activity of LRRK2 kinase. Following this, we induced cellular ER stress responses by subjecting the neurons to different concentrations or durations of tunicamycin treatment. Surprisingly, pre‐treatment with MLi‐2, which effectively inhibited LRRK2 kinase activity, resulted in a heightened sensitivity of cells to tunicamycin. In comparison to the untreated group, MLi‐2‐treated cells exhibited a significant recovery in Ca^2+^ levels (Figure 2G,H). Simultaneously, the inhibition of LRRK2 kinase activity resulted in the restoration of impaired mitochondrial function (Figure 2I).

Furthermore, we conducted additional assessments to determine the level of apoptosis. Interestingly, we found that MLi‐2 treatment significantly rised cell viability and concomitantly reduced the level of apoptosis (Figure 2J,K; Figure S2A,B, Supporting Information). Moreover, we observed that MLi‐2 treatment significantly inhibited the release of ROS in LRRK2 G2019S cells. It suggests that MLi‐2 can potentially mitigate oxidative stress associated with the LRRK2 G2019S mutation (Figure 2L,M).

### 2.3 LRRK2 Interacts with THBS1

To gain further insights into the regulatory mechanism of LRRK2 G2019S, we explored potential downstream targets by searching the publicly available GEO database. By analyzing the database, we aimed to identify genes or factors that may be influenced by or associated with LRRK2 G2019S (**Table** **1**).

**Table 1:** Datasets information.

Database	No.	Published date	Samples
GEO	GSE33298	Submitted in 2011 and updated in 2018	Fibroblast iPSC‐induced neural cells
GEO	GSE36321	2014	H9 hESC derived NSC

**Figure 3:**
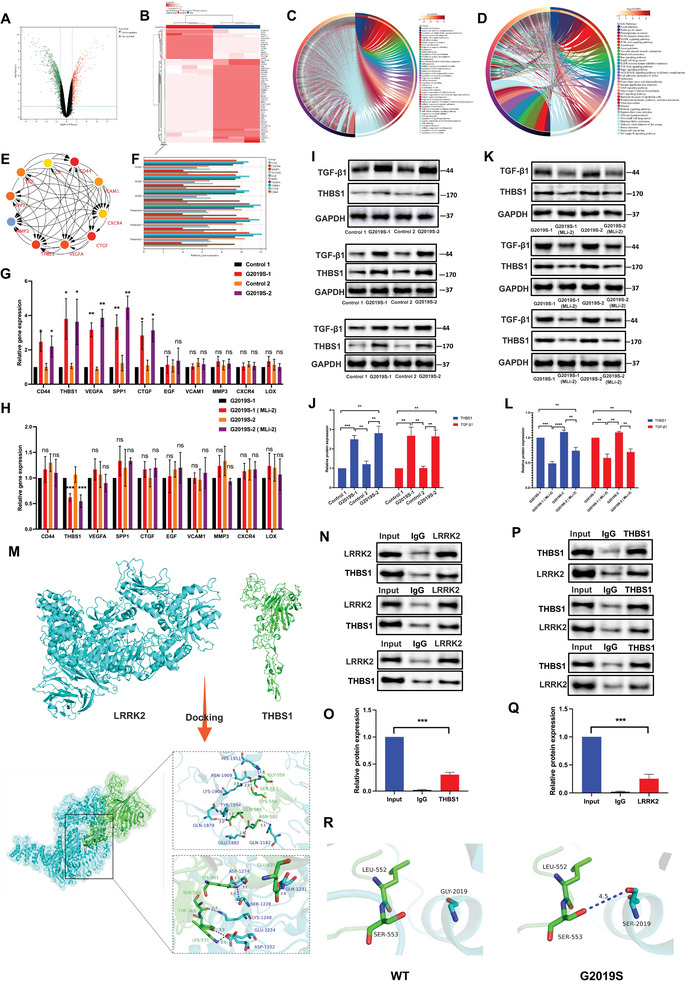
LRRK2 interacts with THBS1. A) Volcano plots were utilized to visually present the DEGs. B) The heat maps of module correlations display samples and genes in each cell, along with *P* values and correlation coefficients. C) The BP category identifies gene list enrichments. As a background enrichment, all genes in the genome were employed. The enrichment factor is the ratio from observed counts to predicted counts. Terms were having a *p*‐value of <0.01, a minimum count of three, and an enrichment factor of >1.5 are aggregated and categorized into clusters based on membership commonality. D) KEGG biological pathways analysis of 607 involved in the mutation of LRRK2. E) The STRING web program created a PPI network for the hub genes to show the interaction between the proteins with the top hub genes. F) The relative hub genes levels of CD44, CTGF, THBS1, VEGFA, SPP1, epidermal growth factor (EGF), vascular cell adhesion molecule 1 (VCAM1), matrix metalloproteinase‐3 (MMP3), C‐X‐C chemokine receptor type 4 (CXCR4), and lysyl oxidase (LOX) in LRRK2 wild and mutation group. G) The relative gene expression levels of CD44, CTGF, THBS1, VEGFA, SPP1, EGF, VCAM1, MMP3, CXCR4, and LOX in LRRK2 were determined using TR‐qPCR in the wild and LRRK2 G2019S groups. The resulting mRNA expression values were normalized to the expression of GAPDH. H. The MLi‐2 compound was employed as a pharmacological inhibitor to target and inhibit the kinase activity of LRRK2. Then the relative gene expression levels of CD44, CTGF, THBS1, VEGFA, SPP1, EGF, VCAM1, MMP3, CXCR4, and LOX in LRRK2 were determined using TR‐qPCR. The resulting mRNA expression values were normalized to the expression of GAPDH. The protein levels of THBS1 and TGF‐β1 proteins were analyzed in triplicate through I) WB analysis and J) their relative expression was calculated. The MLi‐2 compound was employed as a pharmacological inhibitor to target and inhibit the kinase activity of LRRK2. The protein levels of THBS1 and TGF‐β1 proteins were analyzed in triplicate through K) WB analysis and L) their relative expression was calculated. M) Molecular docking was applied to verify the binding activity between LRRK2 and THBS1. The blue color represents the LRRK2 chain, and the green‐blue color represents the THBS1 chain. The blue dashed lines indicate hydrogen bonds, while the red dashed lines represent salt bridge interactions. The negatively charged ASP1274 in LRRK2 forms a salt bridge interaction with the positively charged LYS561 in the THBS1 protein. Additionally, several hydrogen bond interactions are formed between specific amino acids in LRRK2, including ASP1202, GLU1224, LYS1249, SER1228, ASP1274, GLN1182, GLU1882, GLN1879, TYR1894, LYS1906, ASN1909, and HIS1911, and specific amino acids in THBS1, including LYS571, TYR565, SER564, LYS561, GLU635, ASN582, GLN585, CYS556, SER553, and GLY559, which contribute to the stable binding of the two proteins. N–Q) Antibodies specific for LRRK2 and THBS1 were used to immunoprecipitation (IP) and reverse‐IP lysates from DA cells. Western blot analysis of immunoprecipitated proteins was performed using antibodies specific for LRRK2 and THBS1. Data were normalized to GAPDH. R) The protein structure of the wild‐type and G2019S mutant is depicted, displaying the binding interface of THBS1 and LRRK2. Blue corresponds to LRRK2, green represents THBS1, and the sticks indicate the amino acid positions within the proteins. The left side is the wild‐type protein structure, while the right side is the structure of G2019S mutation. Data were presented as means ± SD. The experiments were carried out three times (*n* = 3). One‐way ANOVA followed by Tukey's multiple comparison test in (G, H, J, L, O, Q). The difference in folds is statistically significant. ^*^P < 0.05, ^**^P < 0.01, ^***^ *P* < 0.001.

The GEO database contains two microarray transcriptome datasets related to LRRK2, which were conducted using three different cell lines. Applying a threshold of False Discovery Rate (FDR) ≤ 0.05, we identified a total of 607 differentially expressed genes (DEGs) that were confirmed in these datasets (**Figure** **3**A,B). We utilized the DAVID (Database for Annotation, Visualization, and Integrated Discovery) tool to gain further insights into the biological processes and pathways associated with the DEGs identified. The DEGs were subjected to Gene Ontology (GO) and Kyoto Encyclopedia of Genes and Genomes (KEGG) analyses using DAVID (Table S1, Supporting Information). The 607 genes were primarily enriched in the biological progress (BP) category for system development, regulation of the multicellular organismal process, anatomical structure morphogenesis, nervous system development, cellular developmental process, and cell differentiation (Figure 3C). Many transcription factors were enriched, which have been associated with the LRRK2 mutation (Table S2, Supporting Information). The 607 genes were primarily enriched in focal adhesion, proteoglycans in cancer, extracellular matrix‐receptor interaction, mitogen‐activated protein kinase signaling pathway, phosphatidylinositol 3‐kinase/protein kinase B signaling pathway, amoebiasis, axon guidance, vascular smooth muscle contraction, and rat sarcoma signaling pathway according to KEGG pathway analysis (Figure 3D).

Based on the protein‐protein interaction (PPI) network analysis depicted in Figure S4 (Supporting Information), we identified 10 hub genes and their essential interactions (Figure 3E,F). The hub genes represent key components within the network that play critical roles in mediating interactions and coordinating functions. They are likely to have significant implications in the context of the studied biological process or disease, providing valuable insights into the underlying molecular mechanisms.^[^ ^17^ ^]^ Therefore, we performed RT‐qPCR to validate the expression of the identified hub gene in LRRK2 G2019S DA cells. The findings of our study demonstrated that the levels of CD44, CTGF, THBS1, VEGFA, and SPP1 were significantly upregulated in the LRRK2 G2019S group (Figure 3G). These genes exhibited a promising potential as effectors for distinguishing the underlying pathogenesis of PD. Moreover, our investigation revealed that the inhibition of LRRK2 kinase activity by MLi‐2 resulted in a significant reduction in the expression of THBS1, a hub gene within the network (Figure 3H). By modulating LRRK2 kinase activity, we can potentially influence the expression of THBS1, indicating its involvement in the signaling cascade regulated by LRRK2. We conducted a WB analysis to gain more insights into the expression of THBS1. Consistent with our findings, we observed a notable increase in THBS1 expression in the LRRK2 G2019S mutation group (Figure 3I,J). This further supports the notion that the LRRK2 G2019S mutation contributes to the upregulation of THBS1. Moreover, when we treated the LRRK2 G2019S mutant cells with MLi‐2, we observed a significant reduction in the protein expression level of THBS1 (Figure 3K,L). This implies that the inhibition of LRRK2 kinase activity by MLi‐2 can effectively suppress THBS1 expression in the context of the LRRK2 G2019S mutation.

THBS1 belongs to the thrombospondin family, which serves as vital components of the extracellular matrix.^[^ ^18^ ^]^ THBS1 was first found in platelets, but it has now been revealed in many studies that it plays a crucial role in disease development.^[^ ^19^ ^]^ Moreover, THBS1 is closely associated with the physiological and pathological processes of the nervous system. Recent research suggests that THBS1 and its ligands produced by neurons could offer a potential avenue for enhancing axon regeneration.^[^ ^20^ ^]^ However, the role of THBS1 in the context of PD and the underlying mechanisms remain largely unknown. Indeed, previous publications have reported the regulatory role of THBS1 could change TGF‐β signaling in renal injury.^[^ ^21^ ^]^ Motivated by these findings, we sought to investigate whether the G2019S mutation could influence the expression of TGF‐β. Remarkably, our results demonstrated that the G2019S mutation leads to a significant upregulation of TGF‐β1 expression, which aligns with the observed expression pattern of THBS1 (Figure 3I,J). Moreover, when we employed MLi‐2 to inhibit LRRK2 kinase activity, we observed a significant reduction in the expression of TGF‐β1 (Figure 3K,L). These results suggest that the G2019S mutation enhances the expression of TGF‐β1 and that the inhibition of LRRK2 kinase activity by MLi‐2 can effectively suppress the expression of TGF‐β1. These findings provide further insights into the regulatory relationship between the G2019S mutation, THBS1, and TGF‐β1, highlighting a potential mechanism through which LRRK2 may modulate TGF‐β signaling in the context of PD.

Molecular docking plays a crucial role in structural molecular biology and computer‐assisted drug design. The objective of ligand‐protein docking is to predict the primary binding mode(s) of a ligand when interacting with a protein of known 2 and 3D structure.^[^ ^22^ ^]^ Using molecular docking, the results revealed that the binding energy between LRRK2 protein and THBS1 protein is −123.58 kcal mol^−1^, exhibiting a stable binding interaction (Figure 3M; Figure S11, Supporting Information). In general, a lower binding energy between two proteins indicates a more stable binding interaction. By employing immunoprecipitation, we aimed to investigate the relationship between LRRK2 and THBS1. Encouragingly, our results revealed a direct interaction between LRRK2 and THBS1 (Figure 3N–Q). This finding indicates that LRRK2 and THBS1 can physically bind to each other, suggesting a potential functional association between these two proteins. What's more, amino acids within a 5 Å radius of GLY2019 in LRRK2 were analyzed using PyMOL. The examination revealed that THBS1's LEU552 and SER553 are in close proximity to LRRK2's GLY2019. Following this observation, a mutation analysis was conducted using PyMOL to measure the distance between THBS1's SER553 and LRRK2's GLY2019. As a result, the 2019th position contains a non‐polar, uncharged glycine that does not interact with the surrounding amino acids of the protein in the wild‐type LRRK2 protein. However, in the mutated form (G2019S), this position is altered, replacing the uncharged glycine with a polar nonpositive serine. The hydroxyl group of the mutated LRRK2 protein's side chain forms a weak hydrogen bond with SER553 of the THBS1 protein, which is the amino acid closest to position G2019S. These changes in amino acid interactions around position 2019 are likely to induce functional alterations in the proteins (Figure 3R).

### 2.4 Knocking Down of THBS1 Reduces ER Stress Via Interacting TGF‐β1

**Figure 4:**
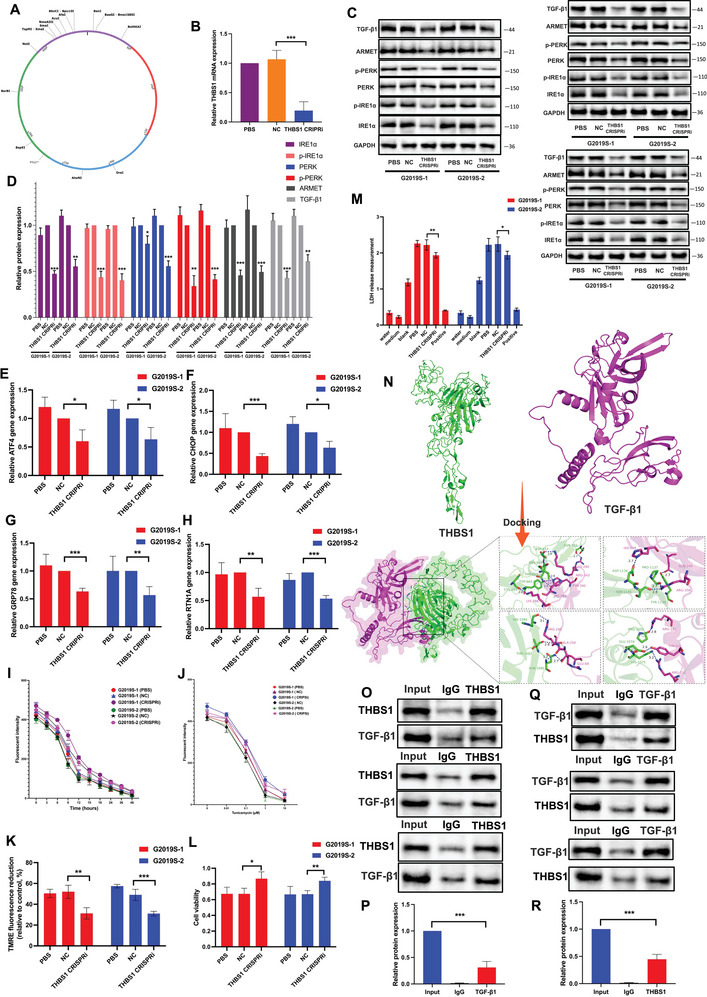
Knocking down of THBS1 reduces ER stress via interacting TGF‐β1. A) Schematic representation of human THBS1 CRIPSRi. B) THBS1 CRIPSRi was transferred into the cells. Knocking down efficiency was confirmed by RT‐qPCR. The resulting mRNA expression values were normalized to the expression of GAPDH. The protein levels of TGF‐β1, ARMET, p‐PERK, PERK, p‐IRE1α, and IRE1α proteins were analyzed in triplicate through C) WB analysis and D) their relative expression was calculated. The intensity of the protein bands was normalized to GAPDH. RT‐qPCR was performed to measure the levels of E) ATF4, F) CHOP, G) GRP78, and H) RTN1A. The resulting mRNA expression values were normalized to the expression of GAPDH. I,J) The DA cells were exposed to Tunicamycin at a concentration of 1 µm for varying time intervals ranging from 0 to 48 h. Additionally, another set of cells was treated with Tunicamycin for 24 h at different concentrations, including 0, 0.01, 0.1, 1, and 10 µm. Fluorescent spectrophotometer traces were recorded, displaying the changes in Mag‐Fluo‐4 AM fluorescence intensity in TG‐treated and CDN‐treated DA cells in comparison to control cells. K) Mitochondrial membrane depolarization in WT and LRRK2 G2019S DA cells was assessed by measuring the fluorescence intensity of TMRE dye at a concentration of 250 nm. L) Cell viability was assessed using the CCK‐8 assay. M) LDH assay was carried out to measure the cell apoptosis. N) Molecular docking was applied to verify the binding activity between THBS1 and TGF‐β1. The purple color represents the TGF‐β1 chain, and the green color represents the THBS1 chain. The blue dashed lines indicate hydrogen bonds. Several hydrogen bond interactions are formed between specific amino acids in THBS1, including ASN601, ASP652, ASN648, THR651, TYR665, CYS663, ASP1134, SER1135, PRO1137, TYR1139, HIS1095, THR1092, ASN1085, PRO1052, GLU1074, and HIS1075, and specific amino acids in TGF‐β1, including LYS280, ARG343, TYR340, HIS283, LYS286, HIS289, GLN349, ARG356, ARG249, ALA250, GLU69, ARG181, TYR1454, and ARG151. These hydrogen bond interactions are crucial for the stable binding of the two proteins. O–R) Antibodies specific for LRRK2 and THBS1 were used to IP and reverse‐IP lysates from DA cells. Western blot analysis of immunoprecipitated proteins was performed using antibodies specific for THBS1 and TGF‐β1. Data were presented as means ± SD. The experiments were carried out three times (*n* = 3). One‐way ANOVA followed by Tukey's multiple comparison test in (B, D, E, F, G, H, K, L, M, P, R). The difference in folds is statistically significant. ^*^P < 0.05, ^**^P < 0.01, ^***^ *P* < 0.001.

To investigate the functional role of THBS1 in the context of LRRK2 mutation‐induced ER stress and TGF‐β1 dysregulation, we employed a specific clustered regularly interspaced short palindromic repeats interference (CRISPRi) approach targeting THBS1 (**Figure** **4**A). We transfected the THBS1 CRISPRi into G2019S DA neurons and assessed the transfection efficacy by performing RT‐qPCR analysis (Figure 4B). Knocking down THBS1 resulted in a significant reduction in the expression and phosphorylation of ER stress‐related proteins, as well as the expression of TGF‐β1 induced by the LRRK2 mutation (Figure 4C,D). Similarly, knocking down THBS1 resulted in significant inhibition of mRNA expression for ATF4, CHOP, RTN1A, and GRP78, indicating that THBS1 plays a crucial role in regulating the expression of essential ER stress‐related genes in the context of LRRK2 mutation (Figure 4E–H).

Furthermore, the CRISPRi‐mediated knockdown of THBS1 resulted in a significant recovery in intracellular Ca^2+^ levels compared to the control group, suggesting that THBS1 may play a role in modulating Ca^2+^ homeostasis in the context of LRRK2 mutation induced‐ER stress (Figure 4I,J). Moreover, the inhibition of THBS1 led to the restoration of impaired mitochondrial function (Figure 4K). Additionally, we observed that the CRISPRi‐mediated knockdown of THBS1 resulted in the rescue of cell viability and a concurrent increase in the level of apoptosis induced by the G2019S mutation (Figure 4L,M). The application of molecular docking analysis revealed a stable binding interaction between the THBS1 protein and the TGF‐β1 protein, with a computed binding energy of −271.6 kcal mol^−1^ (Figure 4N; Figure S12, Supporting Information). Through our immunoprecipitation experiments, we aimed to investigate the relationship between THBS1 and TGF‐β1. Excitingly, our results demonstrated a direct interaction between THBS1 and TGF‐β1 (Figure 4O–R). This finding suggests that THBS1 and TGF‐β1 can form a complex or engage in a molecular interaction within the cellular environment.

### 2.5 Knocking Down of TGF‐β1 Reduces ER Stress

**Figure 5:**
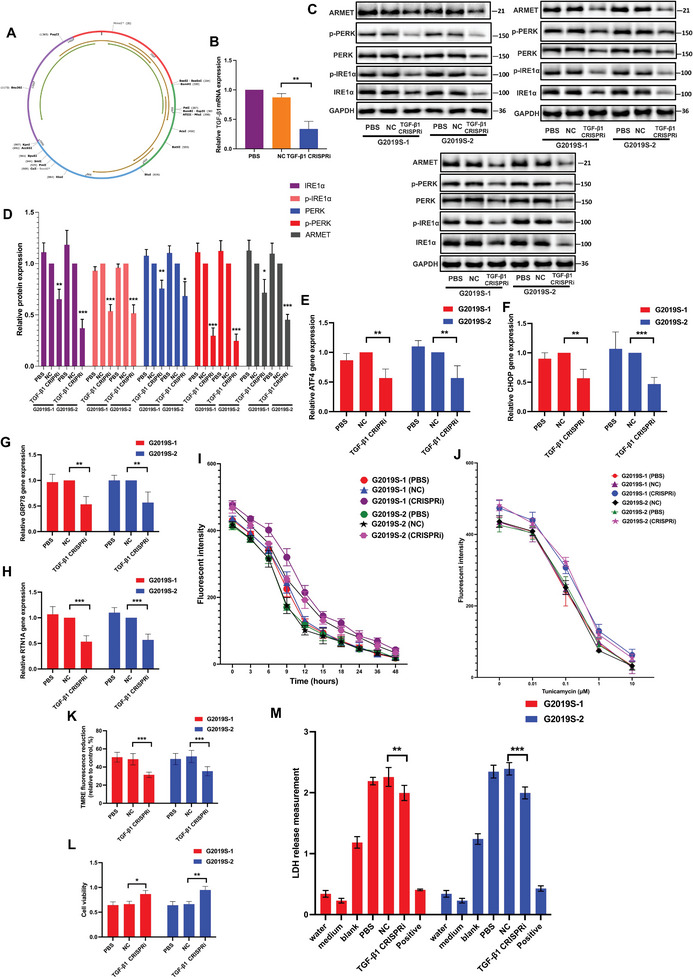
Knocking down of TGF‐β1 reduces ER stress. A) Schematic representation of human TGF‐β1 CRIPSRi. B) The CRISPRi system targeting TGF‐β1 was introduced into the cells to achieve knockdown of TGF‐β1 expression. The knockdown efficiency was confirmed by RT‐qPCR. The resulting mRNA expression values were normalized to the expression of GAPDH. The protein levels of ARMET, p‐PERK, PERK, p‐IRE1α, and IRE1α proteins were analyzed in triplicate through C) WB analysis and D) their relative expression was calculated. The intensity of the protein bands was normalized to GAPDH. RT‐qPCR was performed to measure the levels of E) ATF4, F) CHOP, G) GRP78, and H) RTN1A. The resulting mRNA expression values were normalized to the expression of GAPDH. I,J) The DA cells were exposed to Tunicamycin at a concentration of 1 µm for varying time intervals ranging from 0 to 48 h. Additionally, another set of cells was treated with Tunicamycin for 24 h at different concentrations, including 0, 0.01, 0.1, 1, and 10 µm. Fluorescent spectrophotometer traces were recorded, displaying the changes in Mag‐Fluo‐4 AM fluorescence intensity in TG‐treated and CDN‐treated DA cells in comparison to control cells. K) Mitochondrial membrane depolarization in WT and LRRK2 G2019S DA cells was assessed by measuring the fluorescence intensity of TMRE dye at a concentration of 250 nm. L) Cell viability was assessed using the CCK‐8 assay. M) LDH assay was carried out to measure the cell apoptosis. Data were presented as means ± SD. The experiments were carried out three times (*n* = 3). One‐way ANOVA followed by Tukey's multiple comparison test in (B, D, E, F, G, H, K, L, M). The difference in folds is statistically significant. ^*^P < 0.05, ^**^P < 0.01, ^***^ *P* < 0.001.

Indeed, in our study, we observed that TGF‐β1 may be involved in the process of ER stress induced by LRRK2 G2019S mutation. The expression of TGF‐β1 was significantly increased in cells with the LRRK2 G2019S mutation, which correlated with the induction of ER stress markers. This suggests that TGF‐β1 may be a downstream effector of LRRK2 G2019S and contribute to the development of ER stress in this context. Thus, we utilized a specific CRISPRi approach to knock down TGF‐β1 (**Figure** **5**A). We transfected the TGF‐β1 CRISPRi into dopamine neurons carrying the G2019S mutation and assessed the effectiveness of the transfection by performing RT‐qPCR analysis (Figure 5B). Knocking down TGF‐β1 resulted in a significant reduction in the expression and phosphorylation of ER stress‐related proteins (Figure 5C,D). Similarly, our results revealed that knocking down TGF‐β1 resulted in significant inhibition of mRNA expression for ATF4, CHOP, RTN1A, and GRP78, indicating TGF‐β1 plays a crucial role in regulating the expression of key ER stress‐related genes in the context of LRRK2 mutation (Figure 5E–H).

In addition, our study demonstrated that CRISPRi‐mediated knockdown of TGF‐β1 resulted in a significant recovery in intracellular Ca^2+^ levels compared to the control group (Figure 5I,J). This finding suggests that TGF‐β1 may play a role in modulating intracellular Ca2+ homeostasis in the context of LRRK2 mutation‐induced ER stress. Meanwhile, TGF‐β1 suppression led to the recovery of altered mitochondrial function (Figure 5K). The recovery of cell viability and the contemporary in the level of apoptosis caused by the G2019S mutation were both seen as a consequence of the TGF‐β1 being knocked down using CRISPRi (Figure 5L,M). This finding suggests that TGF‐β1 can regulate ER stress in the context of LRRK2 G2019S mutation‐induced cellular dysfunction.

### 2.6 LRRK2 G2019S could Promote the Expression of THBS1, TGF‐β1, and Induce ER Stress In Vivo

**Figure 6:**
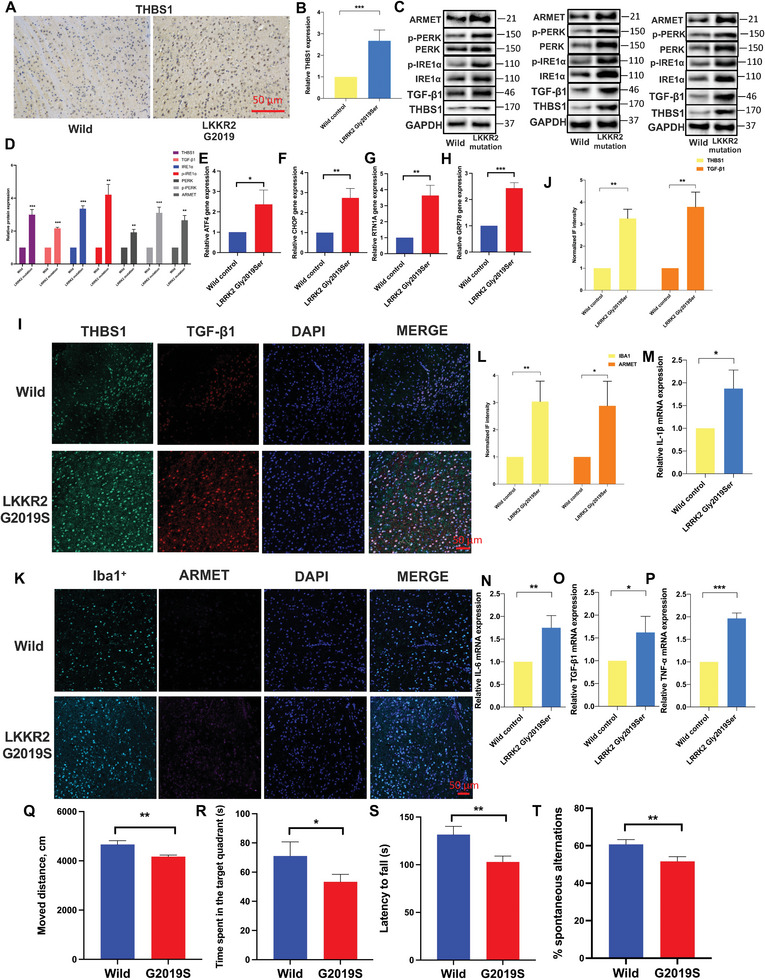
LRRK2 G2019S could promote the expression of THBS1, TGF‐β1, and induce ER stress in vivo. A,B) Immunohistochemical analysis was performed to analyze the THBS1 expression. The protein levels of THBS1, TGF‐β1, ARMET, p‐PERK, PERK, p‐IRE1α, and IRE1α proteins were analyzed in triplicate through C) WB analysis and D) their relative expression was calculated. E‐H. RT‐qPCR was performed to measure the levels of E) ATF4, F) CHOP, G) RTN1A, and H) GRP78. The resulting mRNA expression values were normalized to the expression of GAPDH. I,J) A confocal image provided by immunofluorescence determined the expression levels of THBS1 and TGF‐β1. Green: anti‐THBS1; red: anti‐TGF‐β1. The scale bar represents 50 µm. K,L) A confocal image provided by immunofluorescence determined the expression levels of Iba1^+^ and ARMET. Green: anti‐Iba1^+^; red: anti‐ARMET. The scale bar represents 50 µm. The mRNA expression of M) IL‐1β, N) IL‐6, O) TGF‐β1, and P) TNF‐α were determined by RT‐qPCR. The resulting mRNA expression values were normalized to the expression of GAPDH. Behavioral characterization of mice was analyzed with Q) open‐field, R) Y‐maze, S) Rotarod testing, and T) morris water maze. Data were presented as means ± SD. The experiments were carried out three times (*n* = 3). Unpaired student's *t*‐test in (B, D, E, F, G, H, J, L, M, N, O, P, Q, R, S, T). The difference in folds is statistically significant. ^*^P < 0.05, ^**^P < 0.01, ^***^ *P* < 0.001.

We investigated the impact of LRRK2 G2019S mutation on ER stress and the expression of THBS1 and TGF‐β1 in DA neurons above. We observed that the mutation of LRRK2 significantly promoted ER stress and induced the expression of THBS1 and TGF‐β1 in the neurons. To validate these findings in an in vivo setting, we conducted experiments using LRRK2 G2019S mice. As a result, our findings in LRRK2 G2019S mice were consistent with the in vitro results. We observed a significant increase in the expression of THBS1, TGF‐β1, as well as the expression and phosphorylation of ER stress‐related proteins in the midbrain, specifically in the SNpc, when compared to the wild‐type mice (**Figure** **6**A–D). In addition, we also found a significant increase in the mRNA expression of ATF4, CHOP, RTN1A, and GRP78 in the midbrain of LRRK2 G2019S mice (Figure 6E–H). These results further support the notion that LRRK2 mutation promotes the occurrence of ER stress and dysregulates the expression of THBS1 and TGF‐β1.

In addition to the previous findings, we further employed immunofluorescence staining to confirm that LRRK2 G2019S mutation not only promoted the expression of THBS1 and TGF‐β1, but also resulted in the activation of microglia in the SNpc (Figure 6I–L). Furthermore, we observed a noticeable increase in the mRNA levels of proinflammatory cytokines interleukin‐1 beta (IL‐1β), interleukin‐6 (IL‐6), tumor necrosis factor‐alpha (TNF‐α), and TGF‐β1 in the SNpc of LRRK2 G2019S mice compared to the wild‐type group (Figure 6M–P; Figure S5A–D, Supporting Information). These results suggest that the LRRK2 G2019S mutation may contribute to the upregulation of the proinflammatory cytokines, indicating an inflammatory response in the SNpc region. Next, we conducted a series of behavioral tests, including the Y‐maze, open‐field, rotarod testing, and Morris water maze to assess the behavioral function of the mice. Notably, we observed that the LRRK2 G2019S mutation, significantly aggravated the behavioral burden in vivo (Figure 6Q–T). The mice with the LRRK2 mutation exhibited impaired performance in these tests, indicating deficits in spatial memory, motor coordination, and overall locomotor activity. These findings indicate that the LRRK2 mutation contributes to worsening behavioral functions associated with PD.

### 2.7 Inhibition of THBS1 Attenuates TGF‐β1 and ER Stress Induced by LRRK2 G2019S In Vivo

**Figure 7:**
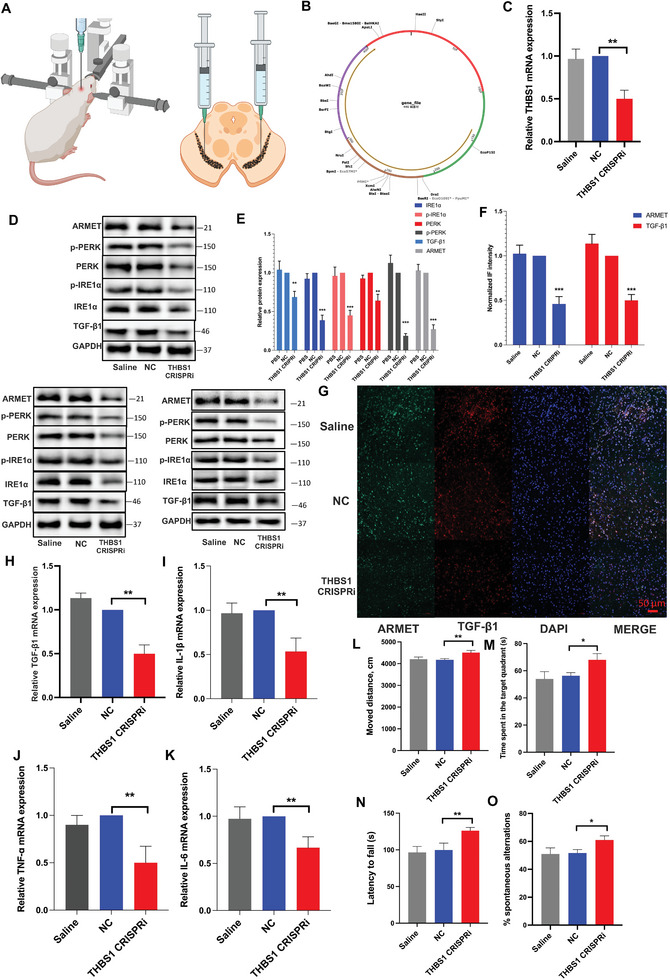
Inhibition of THBS1 attenuates TGF‐β1 and ER stress induced by LRRK2 G2019S in vivo. A) Schematic diagram of stereotaxic injection of plasmids into the SNpc. B) Schematic representation of mice THBS1 CRIPSRi. C) THBS1 CRIPSRi was injected stereotaxically into the SNpc. Knocking down efficiency was confirmed by RT‐qPCR. The resulting mRNA expression values were normalized to the expression of GAPDH. The protein levels of TGF‐β1, ARMET, p‐PERK, PERK, p‐IRE1α, and IRE1α proteins were analyzed in triplicate through D) WB analysis, and E) their relative expression was calculated. The intensity of the protein bands was normalized to GAPDH. F,G) A confocal image provided by immunofluorescence determined the expression levels of TGF‐β1 and ARMET. Green: anti‐ARMET; red: anti‐TGF‐β1. The scale bar represents 50 µm. The mRNA expression of H) TGF‐β1, I) IL‐1β, J) TNF‐α, and K) IL‐6 were determined by RT‐qPCR. The resulting mRNA expression values were normalized to the expression of GAPDH. Behavioral characterization of mice was analysed with L) open‐field, M) Y‐maze, N) Rotarod testing, and O) Morris water maze (O). Data were presented as means ± SD. The experiments were carried out three times (*n* = 3). One‐way ANOVA followed by Tukey's multiple comparison test in (C, E, F, H, I, J, K, L, M, N, O). The difference in folds is statistically significant. ^*^ *P* < 0.05, ^**^ *P* < 0.01, ^***^ *P* < 0.001.

Intrigued by the promising results obtained from knocking down THBS1 to rescue ER stress and cell apoptosis induced by LRRK2 G2019S via TGF‐β1 in DA neurons in vitro, we proceeded to investigate whether the exogenous delivery of THBS1 CRISPRi plasmids could regulate TGF‐β1 and ER stress in vivo (**Figure** **7**A,B). RT‐qPCR was used to assess the knockdown effectiveness (Figure 7C). Consistent with the results observed in vitro experiments, the knockdown of THBS1 in vivo can effectively inhibit the expression and phosphorylation of ER stress‐related proteins induced by the LRRK2 G2019S mutation. Additionally, it can also inhibit the expression of TGF‐β1 (Figure 7D–G). ATF4, CHOP, RTN1A, and GRP78 mRNA expression was reduced when THBS1 was knocked down (Figure S6A–D, Supporting Information).

Meanwhile, we evaluated the effect of THBS1 CRISPRi on microglia activation. Interestingly, suppression of THBS1 expression modulated the activation of microglial cells in the SNpc (Figure S7A,B, Supporting Information). Further, the use of THBS1 CRISPRi resulted in the rescue of enhanced expression of proinflammatory cytokines IL‐1β, IL‐6, TNF‐α, and TGF‐β1, which were induced by the mutation of LRRK2 (Figure 7H–K; Figure S8A–D, Supporting Information). It suggests that THBS1 may play a crucial role in regulating microglial activation and the expression of proinflammatory cytokines in the context of LRRK2 mutation‐induced pathology, most likely by regulating ER stress. In the final assessment of the behavior function of PD mice using Y‐maze, open‐field, rotarod testing, and Morris water maze, the exogenous delivery of THBS1 CRISPRi showed promising results in mitigating the behavioral burden induced by the LRRK2 mutation in mice (Figure 7L–O), indicating targeting THBS1 could have a beneficial effect on improving motor and cognitive functions in the context of LRRK2‐related PD.

### 2.8 TGF‐β1 Suppression Mitigates ER Stress Induced by LRRK2 G2019S In Vivo

**Figure 8:**
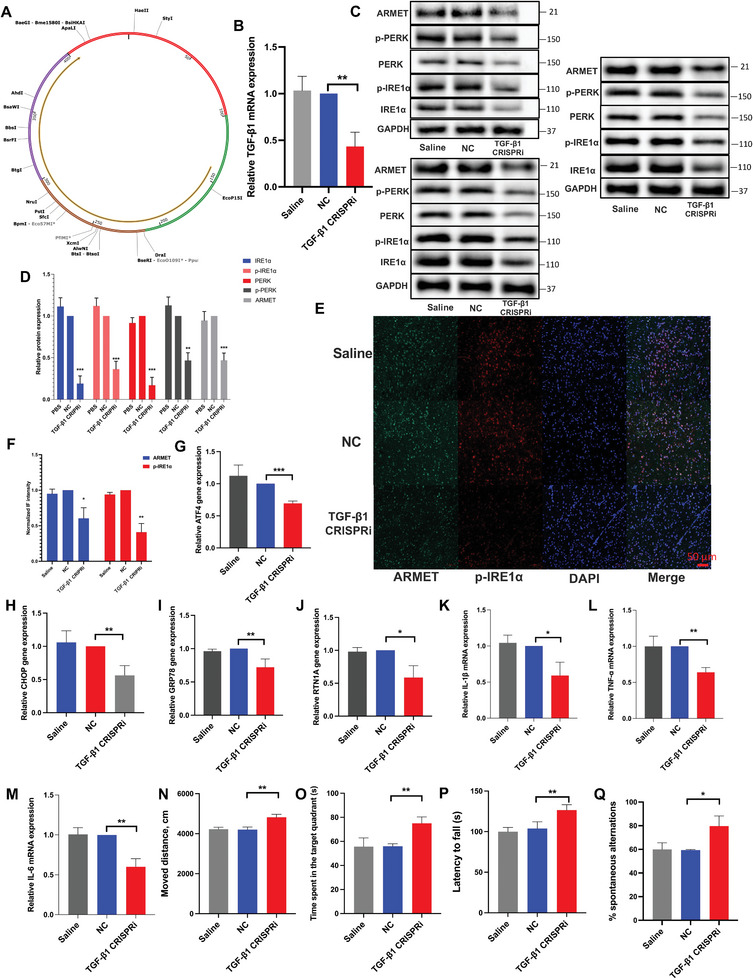
TGF‐β1 suppression mitigates ER stress induced by LRRK2 G2019S in vivo. A) Schematic representation of mice TGF‐β1 CRIPSRi. B) TGF‐β1 CRIPSRi was injected stereotaxically into the SNpc. Knocking down efficiency was confirmed by RT‐qPCR. The resulting mRNA expression values were normalized to the expression of GAPDH. The protein levels of C) ARMET, p‐PERK, PERK, p‐IRE1α, and IRE1α proteins were analyzed through WB analysis and D) their relative expression was calculated. The intensity of the protein bands was normalized to GAPDH. A confocal image provided by immunofluorescence determined the expression levels of E) p‐IRE1α and F) ARMET. Green: anti‐ARMET; red: anti‐p‐IRE1α. The scale bar represents 50 µm. RT‐qPCR was performed to measure the levels of G) ATF4, H) CHOP, I) GRP78, and J) RTN1A. The resulting mRNA expression values were normalized to the expression of GAPDH. The mRNA expression of K) IL‐1β, L) TNF‐α, and M) IL‐6 were determined by RT‐qPCR. The resulting mRNA expression values were normalized to the expression of GAPDH. N–Q) Behavioral characterization of mice was analyzed with L) open‐field, M) Y‐maze, N) Rotarod testing, and O) Morris water maze. Data were presented as means ± SD. The experiments were carried out three times (*n* = 3). One‐way ANOVA followed by Tukey's multiple comparison test in (B, D, F, G, H, I, J, K, L, M, N, O, P, Q). The difference in folds is statistically significant. ^*^ *P* < 0.05, ^**^ *P* < 0.01, ^***^ *P* < 0.001.

After stereotactic intraventricular injection of TGF‐β1 CRISPRi, we evaluated the knocking down efficiency by RT‐qPCR (**Figure** **8**A,B). The inhibition of TGF‐β1 in vivo effectively suppresses the expression and phosphorylation of ER stress‐related proteins induced by the LRRK2 G2019S mutation in vivo (Figure 8C–F). Additionally, TGF‐1 CRISPRi was used to reverse the LRRK2 mutation‐induced increase in the production of ATF4, CHOP, RTN1A, and GRP78 mRNA (Figure 8G–J). These observations suggest that targeting TGF‐β1 can attenuate ER stress in the context of the LRRK2 G2019S mutation.

Subsequently, we observed a significant suppression of microglial activation in the midbrain by inhibiting the expression of TGF‐β1 (Figure S9A,B, Supporting Information). Further, we detected the alteration of pro‐inflammatory cytokines expression; consequently, IL‐1β, IL‐6, and TNF‐α have reduced apparently in midbrain with the injection of TGF‐β1 CRISPRi (Figure 8K–M; Figure S10A–C, Supporting Information). The assessment of behavioral functions in PD mice using various tests, such as Y‐maze, open‐field, rotarod testing, and Morris water maze provides valuable insights into the effects of TGF‐β1 CRISPRi on the behavior improvement of G2019S mice (Figure 8N–Q). The mitigation of behavior burden in PD mice after stereotactic intraventricular injection of TGF‐β1 CRISPRi suggests that targeting TGF‐β1 can positively impact the behavioral symptoms associated with PD.

## 3 Discussion

The LRRK2 G2019S mutation is one of the most common genetic causes of familial PD, contributing to both familial and sporadic forms of the disease.^[^ ^23^ ^]^ However, it is still unclear how LRRK2 kinase activity is regulated at the cellular and molecular levels, as well as how the activity of abnormal LRRK2 kinase results in PD. The pathogenic mechanisms underlying LRRK2 G2019S mutation‐induced neurodegeneration are complex and poorly understood. In this study, we delved into the functional role of THBS1 in the context of LRRK2 mutation‐induced ER stress and TGF‐β1 dysregulation in PD.

The ER is where secreted or membrane‐bound proteins are produced, and it constantly responds to stress signals to regulate overall protein synthesis.^[^ ^8^,^24^ ^]^ The UPR, or simply the ER stress response, is activated by the accumulation of misfolded proteins in the ER. The buildup of misfolded proteins in the brain is a prominent hallmark of most neurodegenerative illnesses, including Alzheimer's disease, amyotrophic lateral sclerosis, Huntington's disease, and PD, according to growing data from recent research.^[^ ^25^ ^]^ Chronic or excessive ER stress can lead to neuronal dysfunction and cell death. Although the role of LRRK2 in ER stress is unknown, research utilizing a C. elegans model missing the LRRK2 homolog showed that LRRK2 was crucial for reducing ER stress and spontaneous neurodegeneration.^[^ ^26^ ^]^ Despite these intriguing findings, the role of ER stress in mutant LRKK2 pathogenic manifestations in mammalian cells has yet to be investigated. In recent years, it has been implicated that ER stress may be vital contributor to LRRK2 G2019S‐related PD pathogenesis.^[^ ^27^ ^]^ However, its pathogenic mechanisms are not fully understood.

Our findings align with recent publications in the field, providing further evidence for the involvement of ER stress in LRRK2‐related PD and highlighting the therapeutic potential of targeting ER stress pathways. First, our study demonstrated that the LRRK2 G2019S mutations promoted ER stress in human DA neurons and LRRK2 G2019S mice. We noted heightened protein expression of ER stress markers because of LRRK2 G2019S mutation, including IRE1α, PERK, and ARMET, alongside significantly increased phosphorylation of p‐PERK and p‐IRE1α. Upon ER stress, these proteins undergo auto‐phosphorylation and activation. The increased protein expression of IRE1α and PERK, as well as their phosphorylated forms (p‐IRE1α and p‐PERK), suggests an active UPR signaling pathway. IRE1α is a type I transmembrane protein involved in ER‐associated protein degradation, protein folding, and lipid synthesis.^[^ ^28^ ^]^ It functions by splicing the mRNA of the transcription factor X‐box binding protein 1 (XBP1).^[^ ^28^ ^]^ Increased protein expression of IRE1α and p‐IRE1α indicates the activation of the IRE1α‐XBP1 pathway, a major branch of the UPR. PERK is another ER stress sensor that regulates protein synthesis. Upon ER stress, PERK phosphorylates eukaryotic initiation factor 2 alpha, which can promote the translation of specific mRNAs, including ATF4, leading to a global decrease in protein synthesis, which helps alleviate the burden on the ER.^[^ ^29^ ^]^ The increased mRNA expression of ATF4 induced by LRRK2 G2019S indicates its upregulation in response to ER stress. ATF4 can activate the expression of various genes, including CHOP.^[^ ^30^ ^]^ CHOP is a transcription factor that contributes to ER stress‐induced apoptosis. The increased mRNA expression of CHOP suggests the activation of apoptosis because of prolonged ER stress induced by LRRK2 mutation. An exciting outcome of our study was the significant increase in apoptosis levels observed in neurons expressing the LRRK2 G2019S mutation. Another gene with increased mRNA expression in LRRK2 G2019S neurons and mice is RTN1A. It participates in the formation of ER tubules and has been implicated in regulating ER stress‐induced apoptosis.^[^ ^31^ ^]^ GRP78 is upregulated to cope with the increased protein load and facilitate proper folding.^[^ ^32^ ^]^ The upregulation of RTN1A and GRP78 mRNA in LRRK2 G2019S neurons and mice suggests an adaptive response to ER stress, which likely modulates ER stress responses. Ca^2+^ plays crucial roles in protein folding, signaling, and apoptosis.^[^ ^33^ ^]^ The ER is an essential intracellular calcium storage organelle. Disturbances in Ca^2+^ homeostasis can exacerbate ER stress and contribute to the activation of the UPR. The observed Ca^2+^ homeostasis imbalance in this study suggests that the ER's ability to regulate calcium levels may be compromised, further implicating the dysregulation of ER function.

Importantly, targeting LRRK2 kinase activity has emerged as a promising therapeutic approach for modulating ER stress in LRRK2‐related PD. In our study, we evaluated the effects of LRRK2 kinase inhibitor MLi‐2 on ER stress in LRRK2 mutant cells. We found that inhibiting LRRK2 kinase activity with MLi‐2 resulted in a reduction in the expression and phosphorylation of ER stress‐related proteins. Moreover, we observed a downregulation of ATF4, CHOP, RTN1A, and GRP78 mRNA expression upon LRRK2 kinase inhibition. ER stress induces ROS production in neurons, leading to cellular damage and neuronal dysfunction. By inhibiting LRRK2 kinase activity, MLi‐2 may interrupt the cascade of events triggered by ER stress and subsequently reduce ROS levels, thereby preserving cellular homeostasis and neuronal viability. Furthermore, our study revealed that MLi‐2 treatment effectively suppressed apoptosis induced by ER stress in LRRK2 G2019S cells. ER stress is known to activate apoptotic pathways; however, treatment with MLi‐2 rescued cells from apoptotic cell death, suggesting that LRRK2 kinase inhibition can confer cytoprotective effects against ER stress‐induced apoptosis. In addition to these effects, our study also revealed that MLi‐2 treatment could rescue Ca^2+^ homeostasis in LRRK2 G2019S cells under ER stress conditions. These findings suggest that targeting LRRK2 kinase activity may hold therapeutic promise for attenuating ER stress in LRRK2‐related PD, reinforcing the potential of LRRK2 kinase inhibitors as valuable therapeutic tools.

We performed and tested a multitiered bioinformatic analysis using the GEO database to investigate global alterations that responded to the mutation of LRRK2. We introduced the recently developed weighted gene co‐expression network analysis (WGCNA) methodology, a commonly used data mining method based on pairwise correlations between variables and is especially useful for investigating biological networks.^[^ ^34^ ^]^ Interaction analysis and prediction were also performed on these genetic variables. A total of 607 DEGs were validated using the FDR ≤ 0.05 criterion. The PPIs network was used to determine the critical hub genes and crucial modules. Finally, the top 10 top hub genes were selected: CD44, CTGF, THBS1, VEGFA, SPP1, EGF, VCAM1, MMP3, CXCR4, and LOX. Moreover, we further validated distinct differences in gene alteration in iPSCs‐induced DA cells. As a result, the expression levels of CD44, CTGF, THBS1, VEGFA, and SPP1 were increased in the LRRK2‐mutated neurons, displaying promising effectors for discriminating the pathogenesis of PD. Furthermore, our investigation delved into the impact of LRRK2 kinase inhibition on the expression of THBS1, which emerged as a hub gene within the PPI network. We utilized MLi‐2, an LRRK2 kinase inhibitor, to suppress LRRK2 kinase activity in the neurons. Remarkably, the inhibition of LRRK2 kinase activity by MLi‐2 resulted in a significant reduction in the expression of THBS1. Therefore, THBS1 emerged as an exciting candidate. In fact, previous papers have shown how THBS1 regulates TGF‐β signaling in relation to renal damage.^[^ ^21^ ^]^ It's interesting to note that the G2019S mutation significantly increases the expression of TGF‐β1. THBS1 is tightly linked to the nervous system's physiological and pathological activities. However, given the limited study investigated THBS1 in PD, further research is warranted to elucidate the precise mechanisms by which THBS1 contributes to disease pathogenesis and explore its potential as a therapeutic target. Therefore, we investigated the impact of inhibiting LRRK2 kinase activity on THBS1 expression. Strikingly, our investigation revealed that the inhibition of LRRK2 kinase activity by MLi‐2 resulted in a significant reduction in the expression of THBS1 and TGF‐β1, and LRRK2 can directly interact with THBS1. These findings suggest that LRRK2 kinase activity can regulate the expression of THBS1 by direct interaction, highlighting a potential link between LRRK2 and THBS1‐related pathways in PD pathogenesis. Meanwhile, the wild‐type LRRK2 protein has a non‐polar, uncharged glycine at the 2019th position, and it does not interact with the surrounding amino acids. However, in the mutated form G2019SER, this position is changed, and a polar, nonpositive serine replaces the glycine. Consequently, the hydroxyl group of the mutated LRRK2 protein's side chain forms a weak hydrogen bond with SER553 of the THBS1 protein, which is the amino acid closest to position 2019. These modifications in the amino acid interactions around position 2019 are likely to lead to functional alterations in the proteins.

In the subsequent steps, we employed a CRISPRi approach to specifically target THBS1 and TGF‐β1 and investigate their effects on ER stress in both the in vivo and in vitro experiments. As a result, knocking down THBS1 and TGF‐β1 emerged as a powerful tool in deciphering the functional consequences of its dysregulation in the context of LRRK2 mutation‐induced ER stress in vivo and in vitro. Our results unveiled a remarkable cascade of events, where THBS1 and TGF‐β1 knockdown led to a significant reduction in the expression and phosphorylation of ER stress‐related proteins. This strengthens the roles of THBS1 and TGF‐β1 as the crucial regulators of ER stress and highlights their potential as therapeutic targets for restoring ER homeostasis in PD. The inhibition of THBS1 and TGF‐β1 also led to the significant suppression of ATF4, CHOP, RTN1A, and GRP78 in vivo and in vitro, key genes involved in the UPR pathway, further underscoring the critical roles of THBS1 and TGF‐β1 in regulating ER stress‐related gene expression in the context of LRRK2 mutation. These findings contribute to our understanding of the molecular mechanisms underlying ER stress in PD and may inform the development of targeted interventions to restore ER homeostasis. Furthermore, our study revealed the impacts of THBS1 and TGF‐β1 on Ca^2+^ homeostasis and cell viability/apoptosis. THBS1 and TGF‐β1 knockdown rescued Ca^2+^ imbalances and improved cell viability while reducing apoptosis in LRRK2 G2019S cells under ER stress conditions. Immunoprecipitation experiments unveiled a direct interaction between THBS1 and TGF‐β1, suggesting the interplay between these molecules in LRRK2‐mutated PD. The association of THBS1 with TGF‐β1 highlights the potential involvement of TGF‐β1 signaling pathways in the pathogenesis of PD and suggests that THBS1‐TGF‐β1 interactions can contribute to disease progression.

Mounting evidence suggests that LRRK2 G2019S mutation plays a role in modulating neuroinflammatory processes.^[^ ^35^ ^]^ Besides, ER stress can activate the UPR and trigger inflammatory signaling pathways, releasing proinflammatory cytokines, and exacerbating neuroinflammation.^[^ ^36^ ^]^ In line with these concepts, we proposed that LRRK2 G2019S‐mediated ER stress may trigger neuroinflammatory responses. We investigated the effects of the LRRK2 G2019S mutation on neuroinflammation and confirmed the activation of microglia in the SNpc of LRRK2 G2019S mutant mice and increased levels of proinflammatory cytokines. Our previous study demonstrated that the excessive activation of microglia can exacerbate the progression of PD.^[^ ^1^, ^37^ ^]^ Hence, microglia activation may play a role in the behavioral impairments associated with LRRK2 G2019S mutation in mice. Moreover, the significant rescue of behavioral impairments caused by LRRK2 G2019S mutation in mice was observed upon inhibiting the expression of THBS1 and TGF‐β1 in the midbrain. This rescue effect could be attributed to the potential improvement of central inflammation associated with ER stress.

**Figure 9:**
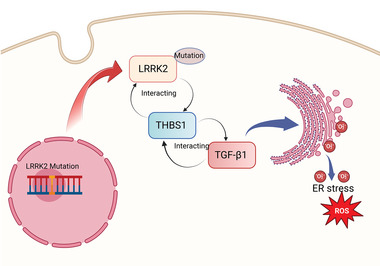
Schematic showing the mechanisms underlying LRRK2 G2019S mutation promotes ER stress via interacting with THBS1/TGF‐β1 in PD.

In conclusion, our study provides valuable insights into the functional roles of THBS1/TGF‐β1 in the context of LRRK2 G2019S mutation‐induced ER stress (**Figure** **9**). Targeting THBS1/TGF‐β1 may hold promise as therapeutic intervention for mitigating ER stress in LRRK2‐related PD.

## 4 Experimental Section

### Reprogramming of Human Fibroblasts to iPSCs and Further Differentiation to DA Neurons

Three samples were taken from two wild healthy volunteers and two from PD patients with the LRRK2 G2019S mutation verified. WT fibroblasts and fibroblasts from PD patients with LRRK2 G2019S mutation were reprogrammed to iPSCs referred to the previous report.^[^ ^38^ ^]^ Three passages were performed to amplify dermal fibroblasts, after which they were reprogrammed utilizing Sendai virus vectors from the Cytotune iPS reprogramming kit (Life Technologies Invitrogen).

Then the iPSCs were cultured in DMEM/F12, 2% of B27 without vitamin A (12587‐010 Gibco), supplemented with 10 µm of Y‐27632 (ROCK inhibitor; Tocris Biochem), 1% of N‐2 Supplement (17502‐048 Gibco), 20 µm of SB431542 (SMAD inhibitor; S4317‐5MG Sigma), 500 ng ml^−1^ of noggin (120–10C Peprotech), and bFGF 2 ng ml^−1^. As previously described, the iPSCs were differentiated into neuroectodermal spheres (NES).^[^ ^38^ ^]^ The NESs were then placed in DA neural patterning media after being cultivated for four days following passaging: B27 supplement (1:50), DMEM/F12, N2 supplement (1:100), 100 ng ml^−1^ FGF8, 100 U ml^−1^ penicillin‐streptomycin (all from Invitrogen), 3 µm CHIR99021 and 100 ng ml^−1^ SHH (Tocris), 1 µm purmorphamine (Tocris), and 0.2 mm 2‐Phospho‐l‐ascorbic acid (Sigma). After four days culture, the medium was changed to terminal differentiation medium: DMEM/F12, N2 supplement (1:100), B27 supplement (1:50), 0.5 mm dibutyryl‐cAMP (Enzo, Farmingdale, NY, USA), 20 ng ml^−1^ BDNF, 100 U ml^−1^ penicillin‐streptomycin 20 ng ml^−1^ GDNF, and 0.2 mm 2‐Phospho‐l‐ascorbic acid (Sigma). The cells were cultured at 37 °C in a humidified incubator with 95% air:5% CO_2_.

### Western Blot

The protocol of WB could be referred to the previous studies.^[^ ^1^, ^39^ ^]^ The total protein was obtained by utilizing the radioimmunoprecipitation assay lysis buffer Beyotime, Jiangsu, China) along with protease and phosphatase inhibitors (BioTools, Olathe, KS, USA), following the instructions provided by the manufacturer.^[^ ^40^ ^]^ The protein concentration was determined using the bicinchoninic acid (BCA) protein assay (Bio‐Rad Laboratories, Inc., Berkeley, CA, USA). Equivalent amounts of protein were separated through sodium dodecyl sulfate‐polyacrylamide gel electrophoresis (Beyotime Biotechnology, Shanghai, China) and subsequently transferred to a polyvinylidene fluoride membrane (Millipore, Bedford, USA). After blocking with 5% Tris‐buffered saline‐Tween, the membrane was subjected to overnight incubation at 4 °C with the primary antibody. The antibodies utilized in this study were as follows: rabbit anti‐THBS1 antibody (Cell Signaling Technology, Danvers, MA, USA); rabbit anti‐mouse IgG (Thermo Fisher Scientific), rabbit Anti‐ARMET antibody (Abcam), rabbit anti‐Thrombospondin 1 antibody (Abcam), rabbit Anti‐p‐PERK (Thermo Fisher Scientific), rabbit Anti‐PERK (Thermo Fisher Scientific), rabbit Anti‐IRE1α (Cell Signaling Technology, Danvers, MA, USA), rabbit Anti‐p‐IRE1α (Abcam).

### Reverse Transcription‐Quantitative Real‐Time Polymerase Chain Reaction

After collecting the cells, TRIzol reagent would extract total RNA as directed by the manufacturer (Invitrogen, Carlsbad, CA, USA). For the quantification of messenger RNA (mRNA), the RNA was reverse transcribed to complementary DNA (cDNA) with a random primer (Sangon Biotech, Shanghai, China) using a Reverse Transcription Kit (RR047A; Takara, Dalian, China), and the mRNA levels were ascertained by RT‐qPCR according to the reported protocols.^[^ ^41^ ^]^ A human or mice GAPDH primer pair served as the control. The *C*_t_ technique ∆∆*C*_t_ method was used to calculate and normalize relative gene expressions. **Table** **2** lists all of the primer sequences that were used.

**Table 2:** Primer sequences used.

Genes	Primer sequences, 5′–3′	Primer sequences, 5′–3′
	Forward	Reverse
**Homo sapiens**		
CD44	CCAGAAGGAACAGTGGTTTGGC	ACTGTCCTCTGGGCTTGGTGTT
CTGF	CTTGCGAAGCTGACCTGGAAGA	CCGTCGGTACATACTCCACAGA
THBS1	GCTGGAAATGTGGTGCTTGTCC	CTCCATTGTGGTTGAAGCAGGC
SPP1	CGAGGTGATAGTGTGGTTTATGG	GCACCATTCAACTCCTCGCTTTC
VEGFA	TTGCCTTGCTGCTCTACCTCCA	GATGGCAGTAGCTGCGCTGATA
EGF	TGTTGGCAGGTGGTGAAGTT	GGGTGGAGTAGAGTCAAGACAG
VCAM1	CGAATGAGGGGACCACATCTA	CGCTCAGAGGGCTGTCTATC
MMP3	TGAGGACACCAGCATGAACC	ACTTCGGGATGCCAGGAAAG
CXCR4	GGGCAGAGGAGTTAGCCAAG	CCACCTTTTCAGCCAACAGC
LOX	CCCTCCTGCTTCCTTTTCACA	GAATGTCACAGCGCACAACA
ATF4	GTTTTGGATTGGTGGGGTGC	GTATTTGCCCCTCCCTGCTT
CHOP	TCCAACTGCAGAGATGGCAG	TCCTCCTCTTCCTCCTGAGC
RTN1A	GCATGCCATTTCTACCAGCTA	CATTGAAGAGAGCGCCAACG
GRP78	GAACGTCTGATTGGCGATGC	GAGTCGAGCCACCAACAAGA
GAPDH	AGAAGGCTGGGGCTCATTTG	AGGGGCCATCCACAGTCTTC
**Mus musculus**		
THBS1	ACCGGTTATATCAGAGTGGTGATG	TGTCTGAGAAGAACACCATTTCCT
TNF‐α	TATGGCTCAGGGTCCAACTC	GGAAAGCCCATTTGAGTCCT
IL‐1β	CTCACAAGCAGAGCACAAGC	CAGTCCAGCCCATACTTTAGG
IL‐6	TTCCATCCAGTTGCCTTCTT	CATTTCCACGATTTCCCAGA
TGF‐β1	GCACGTGGAGCTGTACCA	CAGCCGGTTGCTGAGGTA
ATF4	CCGGAAATTCGTCAACGAGC	ACTGCTGCTGGATTTCGTGA
CHOP	CAGGAGAACGAGCGGAAAGT	GAGGTGATGCCCACTGTTCA
RTN1A	GACGTCTGCTATCCACCTCG	GTGACCAGAGACACCCTGTG
GRP78	TGTGTGTGAGACCAGAACCG	TCGCTGGGCATCATTGAAGT
GAPDH	GGGAAATTCAACGGCACAGT	AGATGGTGATGGGCTTCCC

### Quantifying Calcium Levels

A quantitative method for the high‐throughput and sensitive detection of ER stress was carried out by referring to a previous study.^[^ ^13^ ^]^ This method involved the quantification of ER calcium (Ca^2+^) levels using the low‐affinity calcium dye, Mag‐Fluo‐4 AM. In this study, cells were first seeded in a black clear‐bottom 96‐well plate and allowed to rest for 24 h. The medium was then removed, and cells were washed with Hank's Buffered Saline Solution containing 20 mm Hepes.4‐(2‐Hydroxyethyl)‐1‐piperazine ethanesulfonic acid (HEPES). Next, the cells were incubated in a solution containing 6 µm Mag‐Fluo‐4 AM (Thermo Fisher Scientific) and 2% pluronic acid (v/v; Thermo Fisher Scientific) in the dark at 37 °C for 1 h. Excess dye was washed off using Hank's Buffered Saline Solution, and fluorescenceintensity in live cells was measured using a fluorescent spectrophotometer at a wavelength of 495ex/515em (Molecular Devices, Gemini EM).^[^ ^42^ ^]^ To assess cytosolic Ca^2+^ levels, a high‐affinity ratiometric Ca^2+^ dye, Fura‐2 AM (Thermo Fisher Scientific), was used as described previously.^[^ ^43^ ^]^ Initially, cells were seeded in clear‐bottom white 96‐well plates (Nunc, Denmark) until reaching 80% confluence. After a 24 h resting period, the cells were washed and treated with Fura‐2AM (2 µm) for a duration of 30 min. Subsequently, the cells were washed again and placed in Hanks' balanced salt solution supplemented with 10 mm HEPES for the entire duration of the experiment. Fluorescence intensity measurements were recorded every minute for a total of 30 min, employing a SpectraMax Gemini EM fluorescent spectrophotometer (Molecular Devices, Sunnyvale, CA) with two distinct wavelengths: excitation at 340 nm emission^−1^ at 515 nm and excitation at 380 nm emission^−1^ at 515 nm.

### Mitochondrial Membrane Potential

The cells were seeded in 6‐well plates and carbonyl cyanide‐4‐(trifluoromethoxy)phenylhydrazone  (50 µm) was added to the cells for a brief period of 15 min. Following the indicated treatments, changes in mitochondrial membrane potential were assessed using the TMRE mitochondrial membrane potential assay kit (Abcam). Flow cytometry was employed to analyze the data according to the manufacturer's protocol. The decrease in fluorescence after each treatment was quantified as a percentage relative to untreated cell populations for each genotype. Data analysis was performed using FlowJo software.

### Lactate Dehydrogenase Assay

Colorimetry was used to measure LDH release in the cell medium, as described previously.^[^ ^44^ ^]^ To begin, cells were plated at 5 × 10^4^ cells per well in a 96‐well plate and incubated for 72 h in a 96‐well plate. After that, they were given various treatments, and the media from each group was collected. An LDH cytotoxicity assay kit was used to measure LDH release in the cell medium according to the manufacturer's procedure (Thermo Fisher Scientific, Waltham, MA, USA). Following an incubation period, the plate was read using a microplate reader (Thermo Fisher Scientific, Waltham, MA, USA) to measure the absorbance, reflecting the LDH release into the medium. The absorbance values were then compared between treated and control groups, and the relative LDH release or cytotoxicity was calculated. Statistical analysis could be applied to determine the significance of any observed differences.

### Cell Counting Kit‐8 Assay

The CCK8) assay was used to determine cell viability. Cells were seeded into 96‐well plates at a density of 3000 cells well^−1^ in 100 µl of complete medium and incubated at 37 °C. The next day, 10 µl of CCK8 reagent (Beyotime, Shanghai, China) was added to each well, and the cells were further incubated for 2 h at 37 °C. To ensure a uniform color distribution, it was important to gently mix the plate on an orbital shaker for 1 min before reading it. The optical density value (OD450) was then measured using a Multiskan microplate reader (Thermo Fisher Scientific, Waltham, MA, USA). The assay was performed in triplicate and repeated at least three times.

### Flow Cytometry Assay

The measurement of intracellular levels of ROS was carried out using 2',7'‐dichlorodihydrofluorescein diacetate (DCFH‐DA) dye from Sigma–Aldrich, St. Louis, MO, in the manner previously described.^[^ ^37^ ^]^ The cells were treated with 10 µm DCFH‐DA dye to detect ROS levels and incubated for 30 min in the dark at 37 °C and 5% CO_2_. After incubation, the cells were washed three times with phosphate‐buffered saline (PBS). ROS levels were detected immediately using a flow cytometer's FL1 green fluorescence channel from BD Biosciences, San Jose, CA. The data obtained was in the form of mean fluorescence intensity, which was further analyzed using the FlowJo software program from TreeStar, Ashland, OR. Positive and negative gates were established by assessing the intensity of an unstained control sample.

An annexin V‐fluorescein isothiocyanate/propidium iodide (PI) apoptosis detection kit (Dojindo, Tokyo, Japan) was used to measure the apoptosis of the cells. The cells were pelleted, resuspended in 5 L of fluorescein isothiocyanate‐labeled annexin V (V‐FITC), and stained with 5 L of propidium iodide staining solution. The cells were then incubated at room temperature for 10 min. While being protected from light. Flow cytometry was used to measure the number of apoptotic cells (FACSVerse; Becton Dickinson, CA, CT, USA).

### Data Acquisition and Processing

Datasets were employed from GEO public database (https://www.ncbi.nlm.nih.gov/gds) for the keywords “LRRK2”. The R software was employed for background correction of sample expression matrix, data downgrade, normalization, log_2_ transformation, and probe reannotation. RMA, devtools, AnnoProbe, limma, Biobase, affyPLM, and GEOquery were among the R packages used in this research. The gene's expression level was calculated by averaging the values of these probes if a single gene in the chip corresponded to many probes. Using Fisher's combined probability test, *P* values were pooled, then adjusted for multiple comparison correction by the FDR. FDR ≤ 0.05 was chosen as the significance level.

### Functional Enrichment Analysis

To determine the most highly enriched pathways, DAVID (https://david.ncifcrf.gov/home.jsp) was utilized to run GO and KEGG functional enrichment investigations, indicating the module's potential biological importance. The *P* values were determined using Fisher's exact probability technique to obtain statistically significant gene sets with meaningful functional annotations and signaling pathways. *P* < 0.05 was used as the significant level.

### Animals and Management

Transgenic mice expressing wild‐type or mutant LRRK2 (G2019S) were created according to the reported protocols.^[^ ^45^ ^]^ The First Affiliated Hospital of Nanchang University Ethics Committee approved the animal procedures, which were carried out in strict accordance with the Guide for the Care and Use of Laboratory Animals (National Institutes of Health, Bethesda, MD, USA). PCR amplification was employed to introduce an HA epitope at the C‐terminus of human wild‐type LRRK2. To generate cDNA encoding human (G2019S) LRRK2, oligonucleotide‐directed mutagenesis was carried out. The DNA mutation of LRRK2 was subsequently validated through DNA sequencing.^[^ ^46^ ^]^ The transgene construct was created by inserting the cDNA of HA‐tagged wild‐type or mutant (G2019S) LRRK2 into a cytomegalovirus (CMV) enhancer/platelet‐derived growth factor (PDGF)‐βchain expression vector. The resulting transgene fragment was ≈11 kb in size and consisted of the CMV enhancer, PDGF‐βchain promoter, cDNA of either wild‐type or mutant (G2019S) LRRK2, and a 3′ SV40pA sequence.

The process of stereotactic intraventricular intubation referred to the previous research.^[^ ^1a,b^ ^]^ Regarding the experiment with the exogenous delivery of plasmids into an animal model, the bilateral substantia nigra of the mice was surgically implanted with a stereotactic catheter (Woruide, Shenzhen, China). The stereotactic injection sites were: Anterior‐Posterior (AP): −3.1 mm, medial‐lateral: ±1.2 mm from the midline, and dorsoventral: −4.4 mm. The mice were kept warm (37 °C) until they recovered from surgery (7 days). The mice were then given 1 dose of THBS1 CRISPRi or TGF‐β1 CRISPRi (Shanghai GenePharma, Shanghai, China) (20 nM of ribonucleotide in a total volume of 5 µl) daily for 5 days via the catheter in SNpc. The ventral midbrain containing the SNpc was dissected and kept at −80 °C for further investigation after removing the brain.

### Immunofluorescence Staining

The immunofluorescence staining protocol was followed as described previously.^[^ ^47^ ^]^ In brief, the animals were anesthetized and subjected to transcardial perfusion with PBS, followed by the use of ice‐cold 4% paraformaldehyde. The brains were then postfixed in the same fixative for a duration of 2 days at 4 °C. Subsequently, the brains were cryoprotected by immersion in 30% sucrose for an additional 2 days at 4 °C. Mesencephalic coronal sections (12 µm) were obtained using a freezing microtome from Leica and were mounted on slides coated with poly‐d‐lysine. The sections were then subjected to incubation with the specified primary antibodies. Subsequently, the slices were subjected to incubation with secondary antibodies.

The antibodies used in this study were listed as following: rabbit‐derive TH antibody (1:1000; Novus Biologicals), rabbit Anti‐ARMET antibody (1:50, Abcam), rabbit anti‐Thrombospondin 1 antibody (1:100, Abcam), mice anti‐TGF‐β1 (1:100; Thermo Fisher Scientific), mice‐derived anti‐Iba‐1 (1:500; Wako Pure Chemicals, Tokyo, Japan), rabbit anti‐ARMET (1:50, Abcam), mice anti‐p‐IRE1α (1:100, Thermo Fisher Scientific), goat anti‐mouse IgG conjugated with Alexa Fluor 488 (1:400; Servicebio), Cy3 conjugated goat anti‐rabbit IgG (1:300; Servicebio). Immunoreactivity fluoresced green under an LSM 880 laser scanning confocal microscope (Carl Zeiss, Oberkochen, Germany). ZEN light software was used to capture and analyze confocal images (Carl Zeiss).

### Immunohistochemical Analysis

The mice were anesthetized with pentobarbital and then perfused with saline, followed by a solution containing 4% polyformaldehyde‐hydrochloric acid. The midbrains were then chosen for further analysis. Immunostaining was carried out referring to the methods as described previously.^[^ ^1^, ^48^ ^]^ In brief, the fixed tissues underwent a series of steps, including dehydration, clearing using ethanol and xylene, and finally embedding in paraffin. The brains were sectioned into coronal sections with a thickness of 5 µm and intervals of 100 µm. The tissue samples were deparaffinized using xylene, followed by rehydration through descending concentrations of ethanol and rinsing with PBS. Heat‐induced antigen retrieval was performed, and then the specimens were incubated with a solution of bovine serum albumin (BSA) (0.01 g BSA + 1000 µl PBS + 10 µl Triton X) for 30 min. Each tissue section was then placed in a hydrogen peroxide solution (H_2_O_2_, 0.03% in PBS) for 15 min in a dark chamber to block endogenous peroxidase. After washing with PBS, the sections were incubated with goat serum for 20 min. Finally, the sections were incubated with the primary antibody overnight at 4 °C. On the following day, the sections were washed three times with PBS for 15 min each and exposed to the secondary antibody. The integrated optical density of THBS1 was measured using Image‐Pro Plus software (Media Cybernetics, Silver Spring, USA). The antibody utilized for this study was: mouse Anti‐THBS1 (1:100, Thermos Fisher Scientific); goat anti‐mouse IgG secondary antibody (Abcam).

### Transfection

CRISPR/Cas9 repression plasmids were designed by CHOPCHOP (https://chopchop.cbu.uib.no/) and synthesized by Shanghai Shenggong Trade Co., Ltd. DNA sequences verified them. The plasmids were transfected using Lipofectamine 3000 (Invitrogen) according to the manufacturer's instructions. RT‐qPCR identified repression efficiencies.

### Molecular Docking

The online platform HDOCK (http://h‐dockphys.hust.edu.cn/) was employed as the molecular docking program for this study. HDOCK was utilized to analyze different conformations of protein‐protein docking, binding activity under different conformations, and interactions between amino acid residues within a distance of 5 Å. The 3D structure of the docking protein THBS1 was obtained from the Alphafold protein database, and non‐redundant protein structures were selected. The structure of LRRK2 was obtained by searching the Protein Data Bank (PDBID: 7li4). PyMOL software (version 2.3.0) from https://pymol.org was used to separate the original ligands and protein structures, perform dehydration, and remove organic molecules. The Discovery Studio software's “prepare” module was utilized for protein preparation, including hydrogenation and protonation. The Ligplus software was employed to analyze the 2D forces between the two proteins. The Discovery Studio software's “analysis interface” module was used to investigate the protein‐protein interaction interface. PyMOL software (version 2.3.0) was used to visualize the amino acid residues involved in the interactions between the two proteins.

### Immunoprecipitation Assay

The immunoprecipitation assay protocol was followed as described previously.^[^ ^49^ ^]^ The cells were lysed using an immunoprecipitation buffer consisting of 1% Triton X‐100, 150 mm NaCl, 10 mm NaH_2_PO_4_, 15 mm Na_2_HPO_4_, 50 mm NaF, 1 mm EDTA, and 1 mm Na_3_VO_4_. Following the incubation of cell lysates with primary antibodies, they were subsequently incubated with Protein G agarose beads (20 µl per reaction, Millipore, Billerica, MA, USA). The beads were washed using immunoprecipitation buffer and then boiled in 2× sample buffer. The immunoprecipitated proteins were identified through western blot analysis. The antibodies in this experiment were as followings: rabbit anti‐Thrombospondin 1 antibody (Abcam), rabbit anti‐LRRK2 antibody (Cell Signaling Technology, Danvers, MA, USA), rabbit anti‐TGF‐β1 antibody (Abcam).

### Behavior Test

The open‐field test lasted 2 h and was conducted on a square open field (100×100 cm) with 50‐centimeter‐high walls and a video‐tracking system (Ethovision XT 7, Noldus Info. Tech., Wageningen, The Netherlands). In the Y‐maze, the percent spontaneous alternation was computed as 100 x [number of alternations / (total arm entries 2)], where arm entrance was defined as placing all four paws inside an arm. The Morris water maze test was applied to measure the cognitive functioning of LRRK2 G2019S mice, and the time spent in the target quadrant (s) was calculated. In rotarod testing, the mice were placed on a rotating rod that gradually increased in rotation speed from 4 to 40 revolutions per minute, following a programmable acceleration paradigm utilizing equipment from NBT Company (San Diego, CA). The duration for which the mice remained on the rotating rod was recorded, with a maximum measurement time of 300 s.

### Statistical Analysis

The results were provided as the mean SD of three separate studies. A two‐tailed Student's *t*‐test and ANOVA were used in the statistical analysis. A statistically significant difference was defined as one with *P* < 0.05.

## Conflict of Interest

The authors declare no conflict of interest.

## Author Contributions

Y.L.P., L.G.H., S.T., and Z.S.Z. conceived and designed the experiments. Y.L.P. performed the literature search, analyzed the data, and predominantly contributed to the writing of the article. Y.L.P., W.B.Y., L.F.F., and Z.Z.J. organized the validation experiment. Guangdong Provincial Key Laboratory on Brain Function Repair and Regeneration provided experimental technical support. Z.S.Z., L.G.H., and S.T. gave the supervision and revised the paper. All authors read and approved the final manuscript.

## Supporting information

Supporting Information

Supplemental Table 1

Supplemental Table 2

## Data Availability

The data that support the findings of this study are available from the corresponding author upon reasonable request.
