# Supplementary material for: LRRK2 Gly2019Ser Mutation Promotes ER Stress via Interacting with THBS1/TGF‐β1 in Parkinson's Disease
Source: Adv Sci (Weinh). 2023 Sep 6;10(30):2303711. doi: 10.1002/advs.202303711 (PMC10602550; doi:10.1002/advs.202303711)
Supplement: Supplementary file 1 — Supporting Information [file ADVS-10-2303711-s003.pdf]

ADVANCED SCIENCE
Open Access

Supporting Information

for *Adv. Sci.*, DOI 10.1002/advs.202303711

LRRK2 Gly2019Ser Mutation Promotes ER Stress via Interacting with THBS1/TGF-β1 in Parkinson's Disease

Longping Yao, Fengfei Lu, Sumeyye Koc, Zijian Zheng, Baoyan Wang, Shizhong Zhang*, Thomas Skutella* and Guohui Lu**

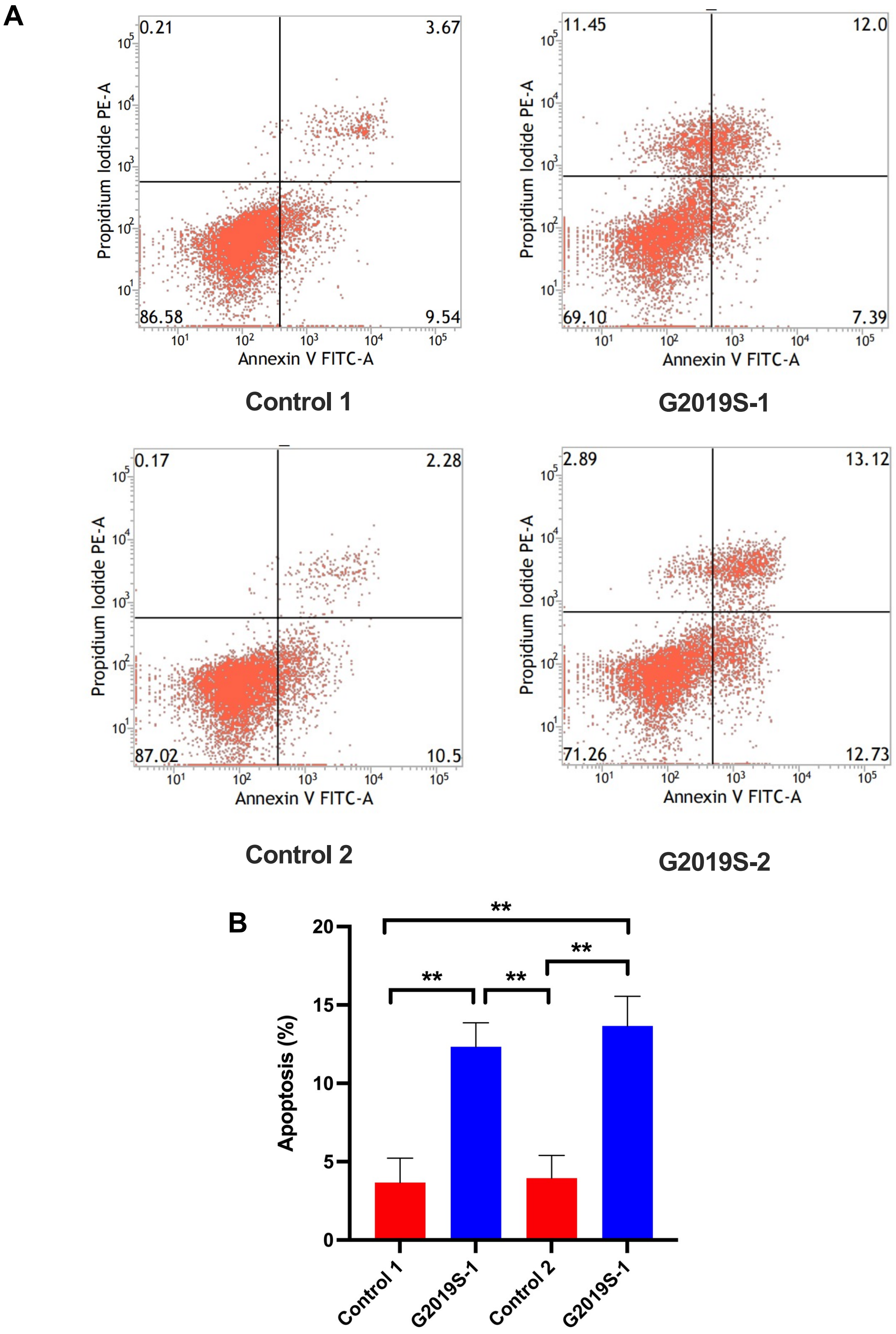

Figure S1. The neuronal apoptosis level was assessed using flow cytometry analysis (a), and the percentage of apoptotic cells in the total neuronal population was calculated (b). Data were presented as means ± sd. The experiments were carried out three times (n=3). One-way ANOVA followed by Tukey's multiple comparison test in B. The difference in folds is statistically significant. ***P* < 0.01.

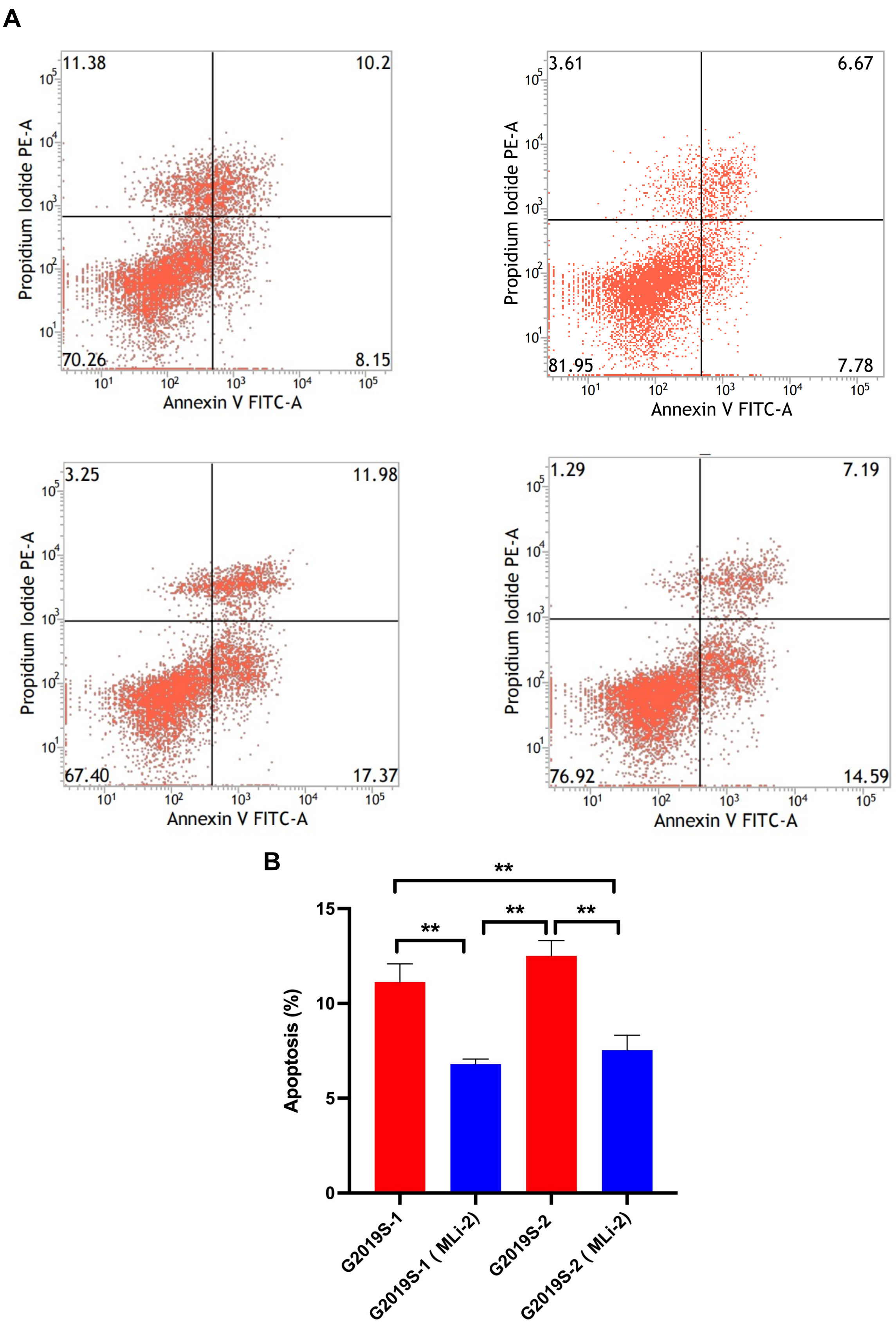

Figure S2. The neuronal apoptosis level was assessed using flow cytometry analysis (a), and the percentage of apoptotic cells in the total neuronal population was calculated (b). Data were presented as means ± sd. The experiments were carried out three times (n=3). One-way ANOVA followed by Tukey's multiple comparison test in B. The difference in folds is statistically significant. $^{**}P < 0.01$.

Figure S3. WGCNA evaluated the genes that had varied expression patterns.
Different databases contribute to the overall number of genes differently, with varying degrees of database overlap (**Supplementary Figure 3A and 3B**) **(Supplementary Table 1)**. The 607 DEGs were hierarchically classified into 18 categories using WGCNA analysis, respectively: brown2, firebrick4, darkgreen, coral2, greenyellow, lightsteelblue, darkolivegreen4, darkred, darkolivegreen, brown4, yellow4, darkseagreen4, plum2, lightyellow, lightcoral, darkorange, royalblue, darkgrey, and black (**Supplementary Figure 3C**). The horizontal axis was the network's average connectivity **(Supplementary Figure 3D)**, and the vertical axis was the scale-free topology fitting index R^2 (the values in the SFT.R.Sq column in the statistical data) **(Supplementary Figure 3E)**. As a consequence of respringing the cluster tree in the heat map, we detected 18 modules of association between module attributes and the tree diagram of gene expression (**Supplementary Figure 3F**). The relationship of modules and a phenotype heat map was exhibited in the grouping of LRRK2 wild and mutation. The blue module showed the most robust inverse link with the LRRK2 mutation phenotype. (**Supplementary Figure 3G**).

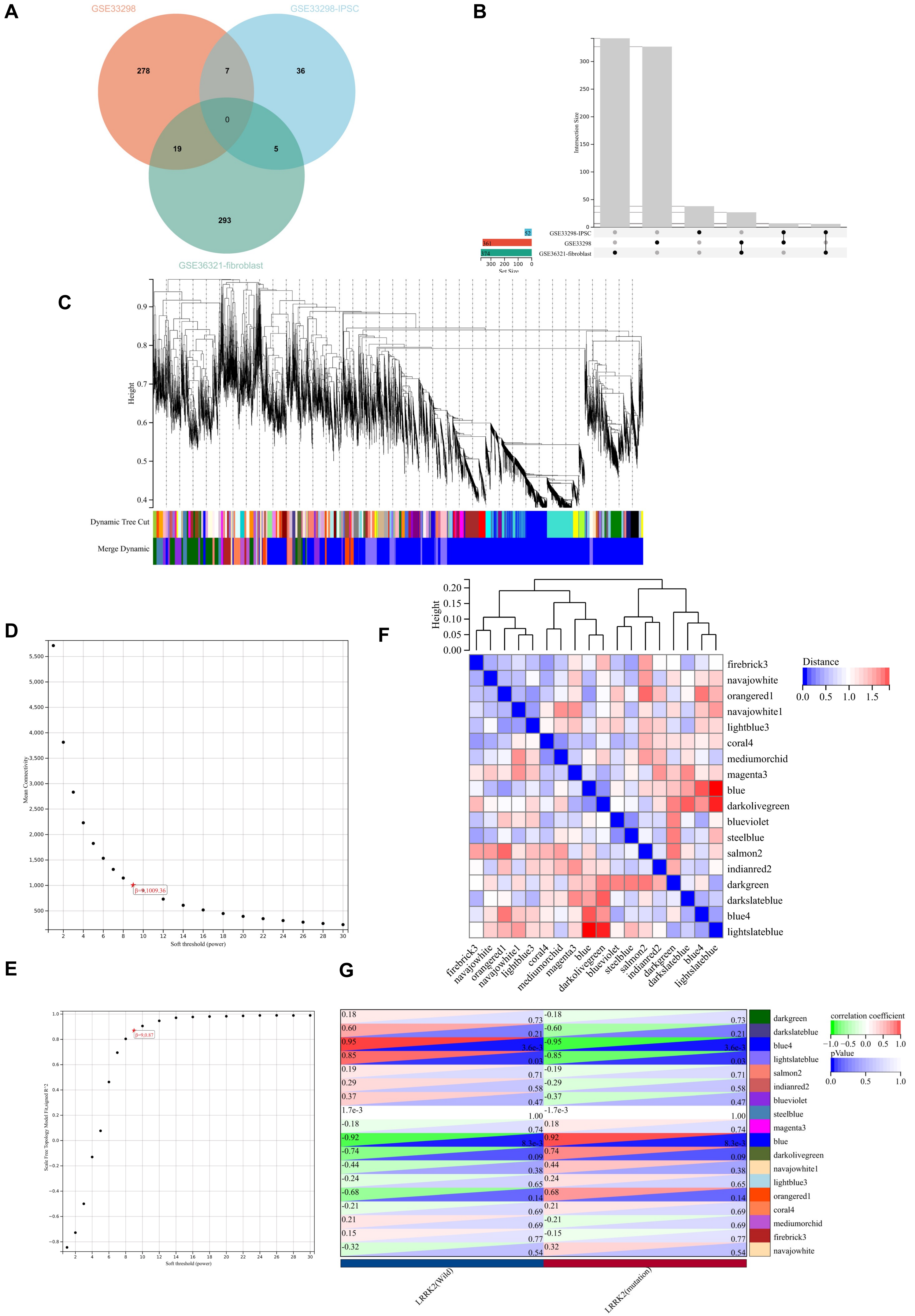

A
GSE33298
GSE33298-IPSC
GSE36321-fibroblast
278
7
36
0
19
5
293
B
Intersection Size
GSE33298-IPSC
GSE33298
GSE36321-fibroblast
52
361
374
300 200 100 0
Set Size
C
Height
Dynamic Tree Cut
Merge Dynamic
D
Mean Connectivity
Soft threshold (power)
β=9,1009.36
E
Scale Free Topology Model Fit,signed R^2
Soft threshold (power)
β=9,0.87
F
Height
Distance
firebrick3
navajowhite
orangered1
navajowhite1
lightblue3
coral4
mediumorchid
magenta3
blue
darkolivegreen
blueviolet
steelblue
salmon2
indianred2
darkgreen
darkslateblue
blue4
lightslateblue
G
correlation coefficient
pValue
darkgreen
darkslateblue
blue4
lightslateblue
salmon2
indianred2
blueviolet
steelblue
magenta3
blue
darkolivegreen
navajowhite1
lightblue3
orangered1
coral4
mediumorchid
firebrick3
navajowhite
LRRK2(Wild)
LRRK2(mutation)

Figure S3. WGCNA analysis the genes which expressed differentially. A-B. The Venn diagrams of the four datasets. **C.** A tree of all gene expressions was created using 1-TOM cluster. **D.** The weight is represented by Soft Threshold (Power), while the vertical axis shows the network's average connectivity. **E.** Soft Threshold (Power) stands for the weight, and the scale-free topology fitting index R^2 is indicated on the vertical axis. **F.** The heat maps of module correlations display samples and genes in each cell, along with *P* values and correlation coefficients. **G.** Genes and samples are classified into PD and control correlations between modules in the heat maps, with correlation coefficients and *P* values in each cell.

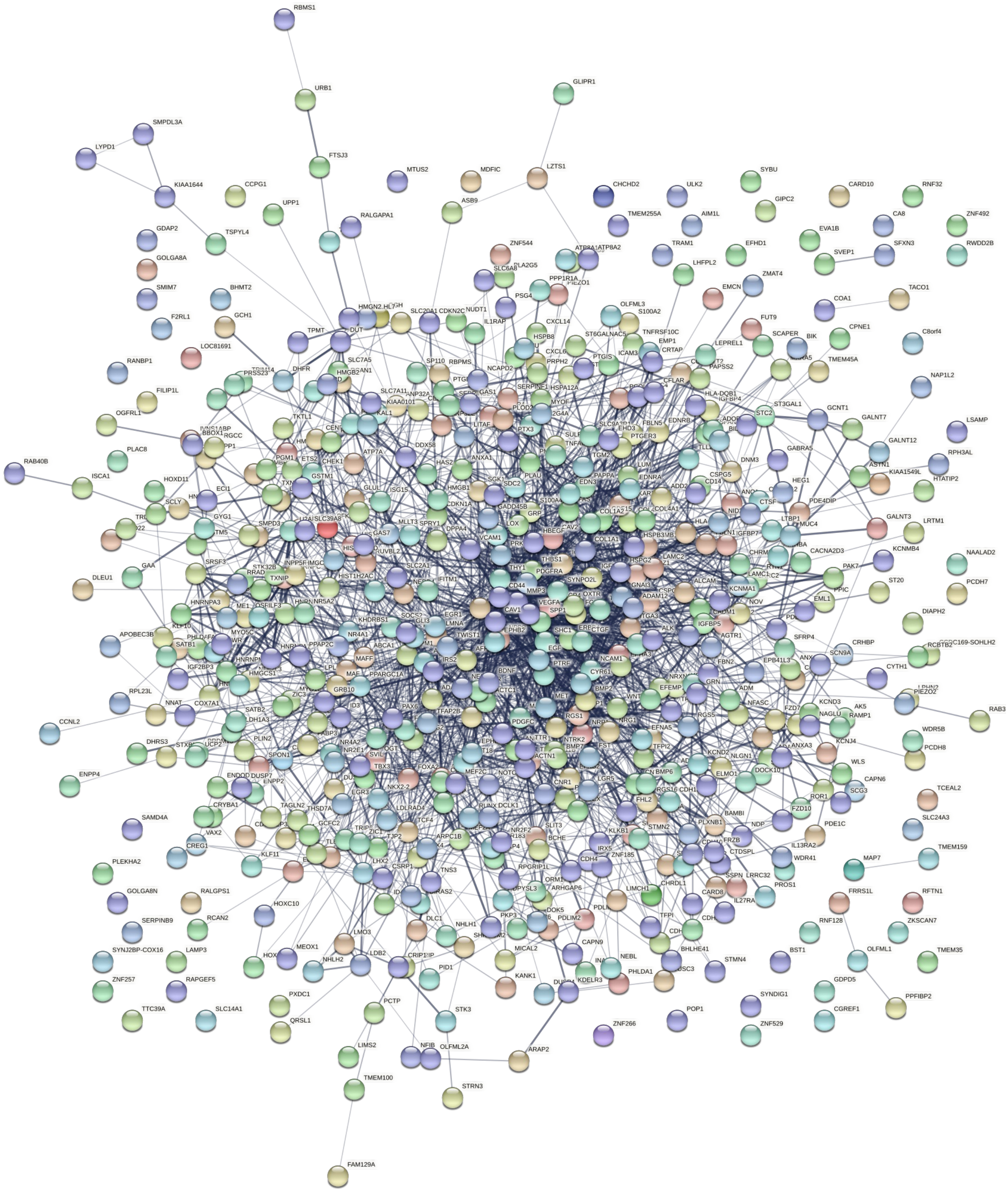

Figure S4. The PPI network reveals interacting proteins. The STRING online tool constructed a PPI network for the genes in DEGs. There were more than 600 protein-protein interactions.

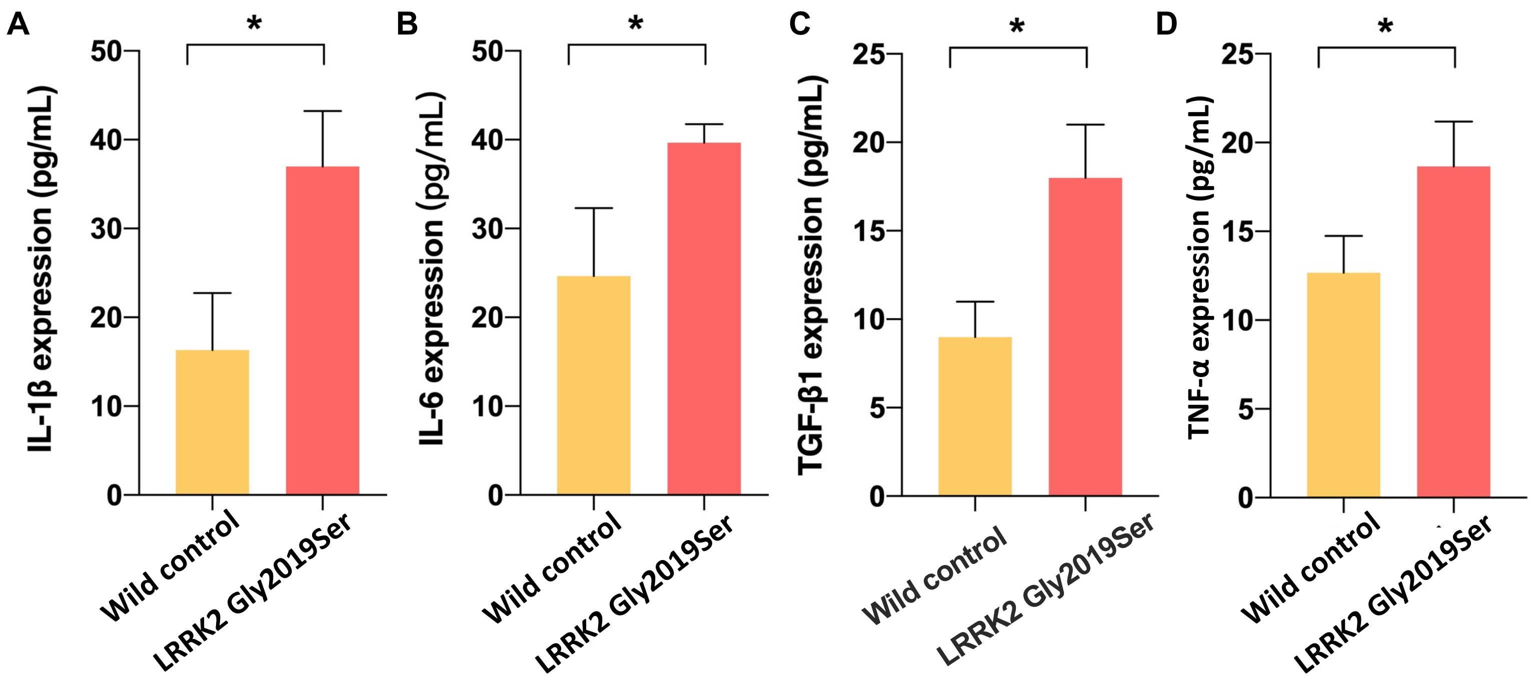

Figure S5. ELISA assay demonstrated the protein levels of IL-1β (A), IL-6 (B), TGF-β1 (C), and TNF-α (D) released to the medium. Data were presented as means ± sd. The experiments were carried out three times (n=3). Unpaired student's t-test in (A, B, C, D). The difference in folds is statistically significant. $*P < 0.05$.

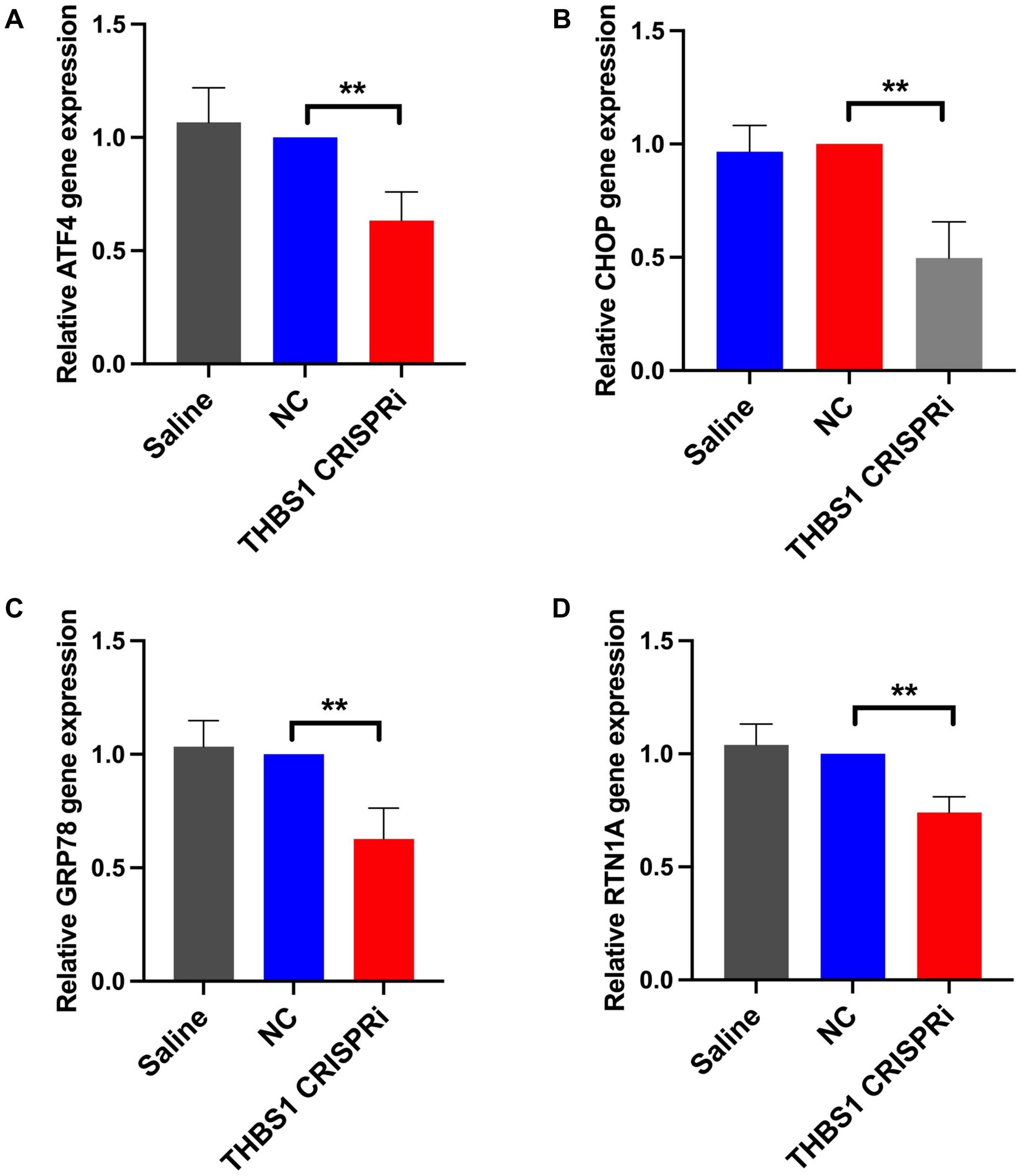

Figure S6. The mRNA expression of ATF4 (A), CHOP (B), RTN1A (C), and GRP78 (D) were determined by RT-qPCR. Data were presented as means ± sd. The experiments were carried out three times (n=3). One-way ANOVA followed by Tukey's multiple comparison test in (A, B, C, D). The difference in folds is statistically significant. ***P* < 0.01.

A

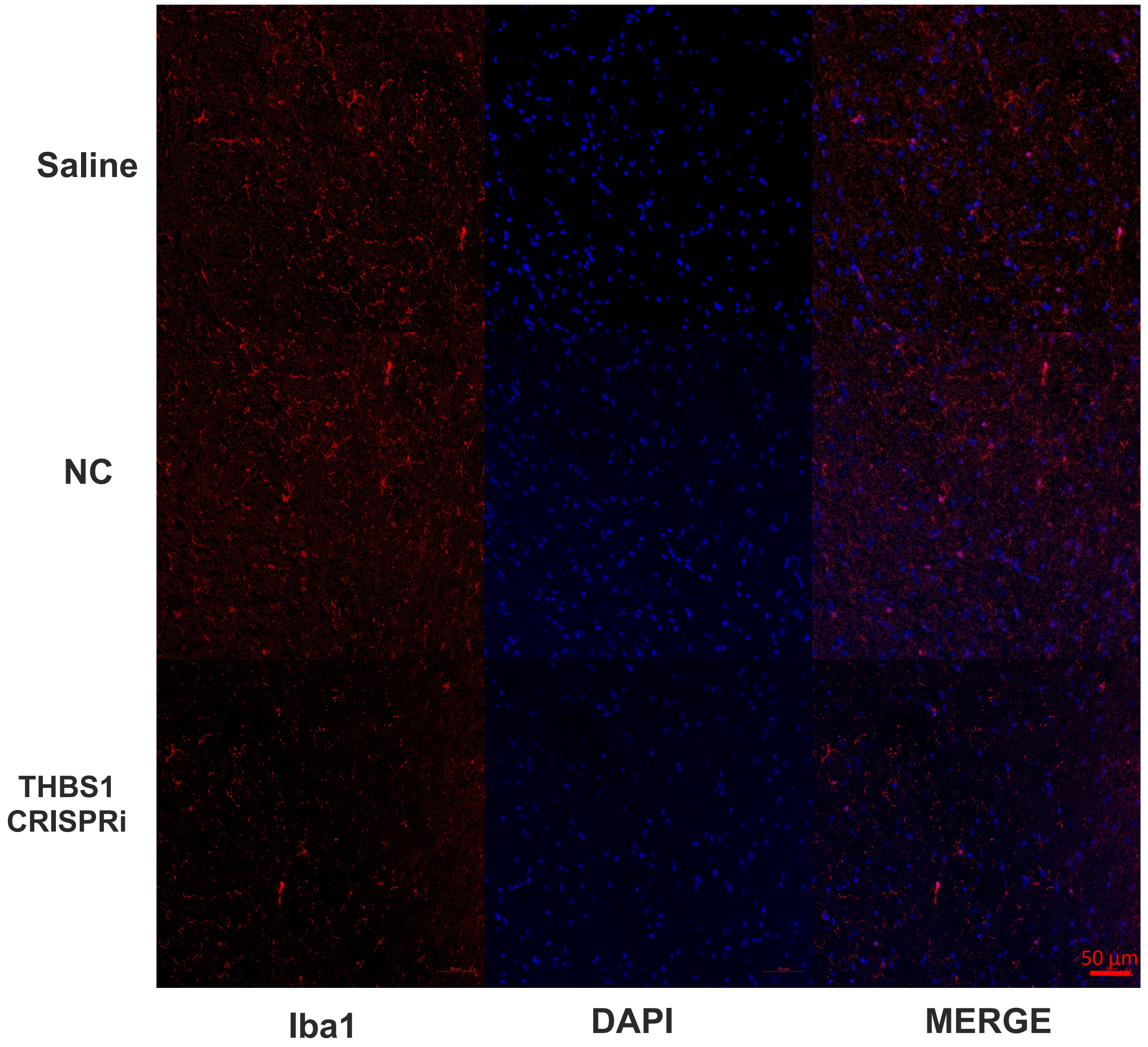

B

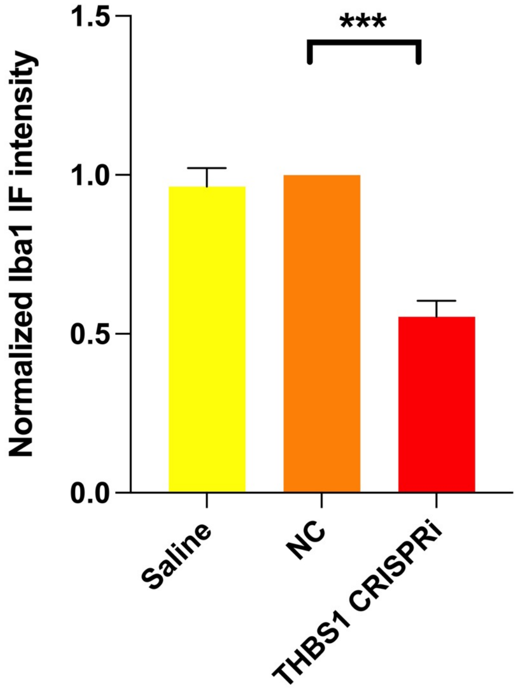

Figure S7. (A, B) A confocal image provided by immunofluorescence determined the expression levels of Iba1+. Red: anti-Iba1. The scale bar represents 50 μm. Data were presented as means ± sd. The experiments were carried out three times (n=3). One-way ANOVA followed by Tukey's multiple comparison test in B. The difference in folds is statistically significant. ****P* < 0.001.

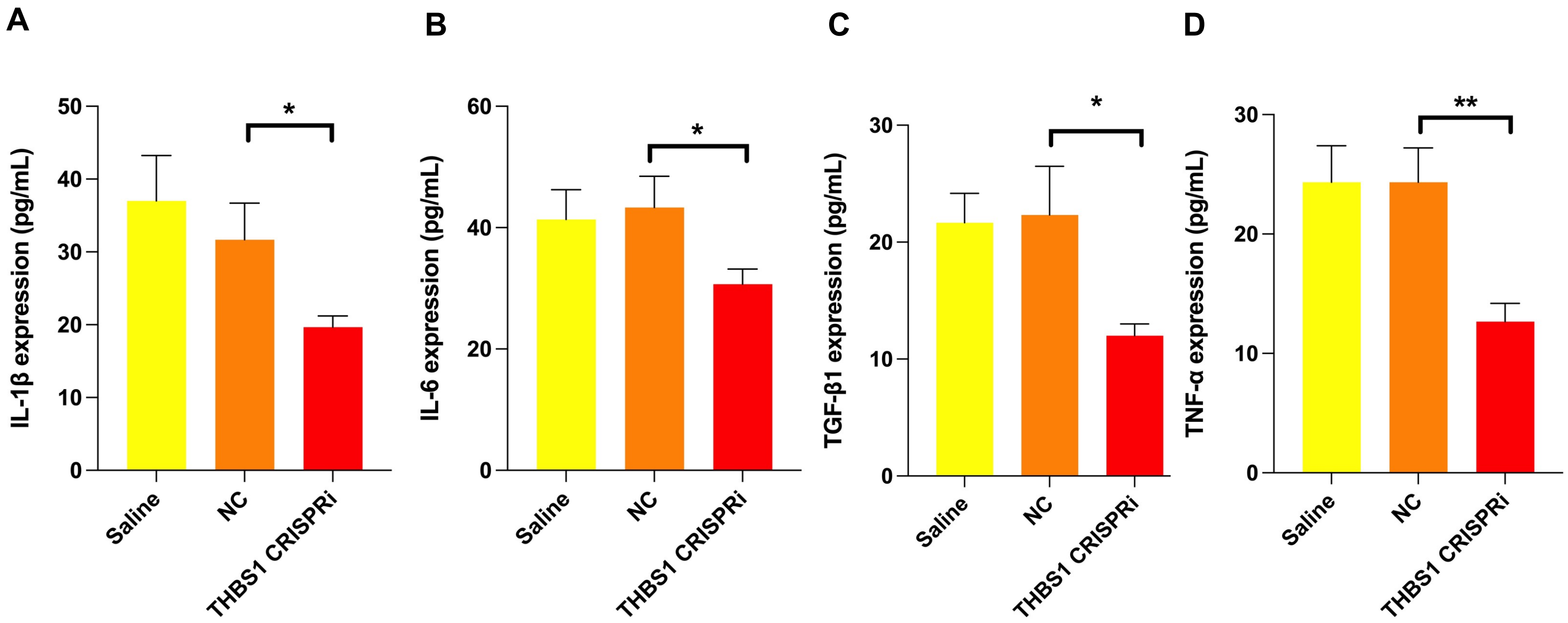

Figure S8. ELISA assay demonstrated the protein levels of IL-1β (A), IL-6 (B), TGF-β1 (C), and TNF-α (D) from midbrain of mice. Data were presented as means ± sd. The experiments were carried out three times (n=3). One-way ANOVA followed by Tukey's multiple comparison test in (A, B, C, D). The difference in folds is statistically significant. *$P < 0.05$, **$P < 0.01$.

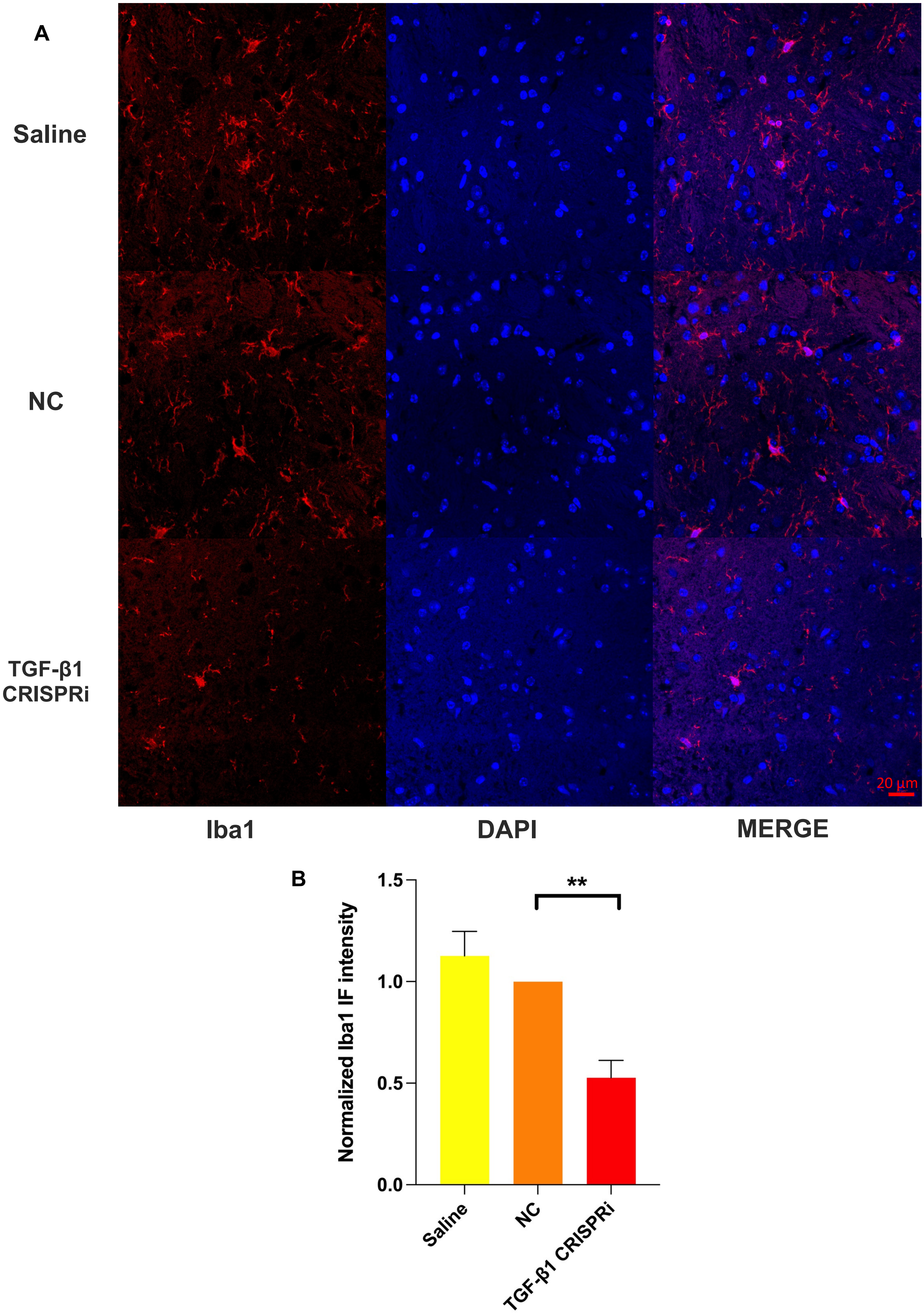

Figure S9. (A, B) A confocal image provided by immunofluorescence determined the expression levels of Iba1+. Red: anti-Iba1. The scale bar represents 20 μm. Data were presented as means ± sd. The experiments were carried out three times (n=3). One-way ANOVA followed by Tukey's multiple comparison test in B. The difference in folds is statistically significant. $^{**}P < 0.01$.

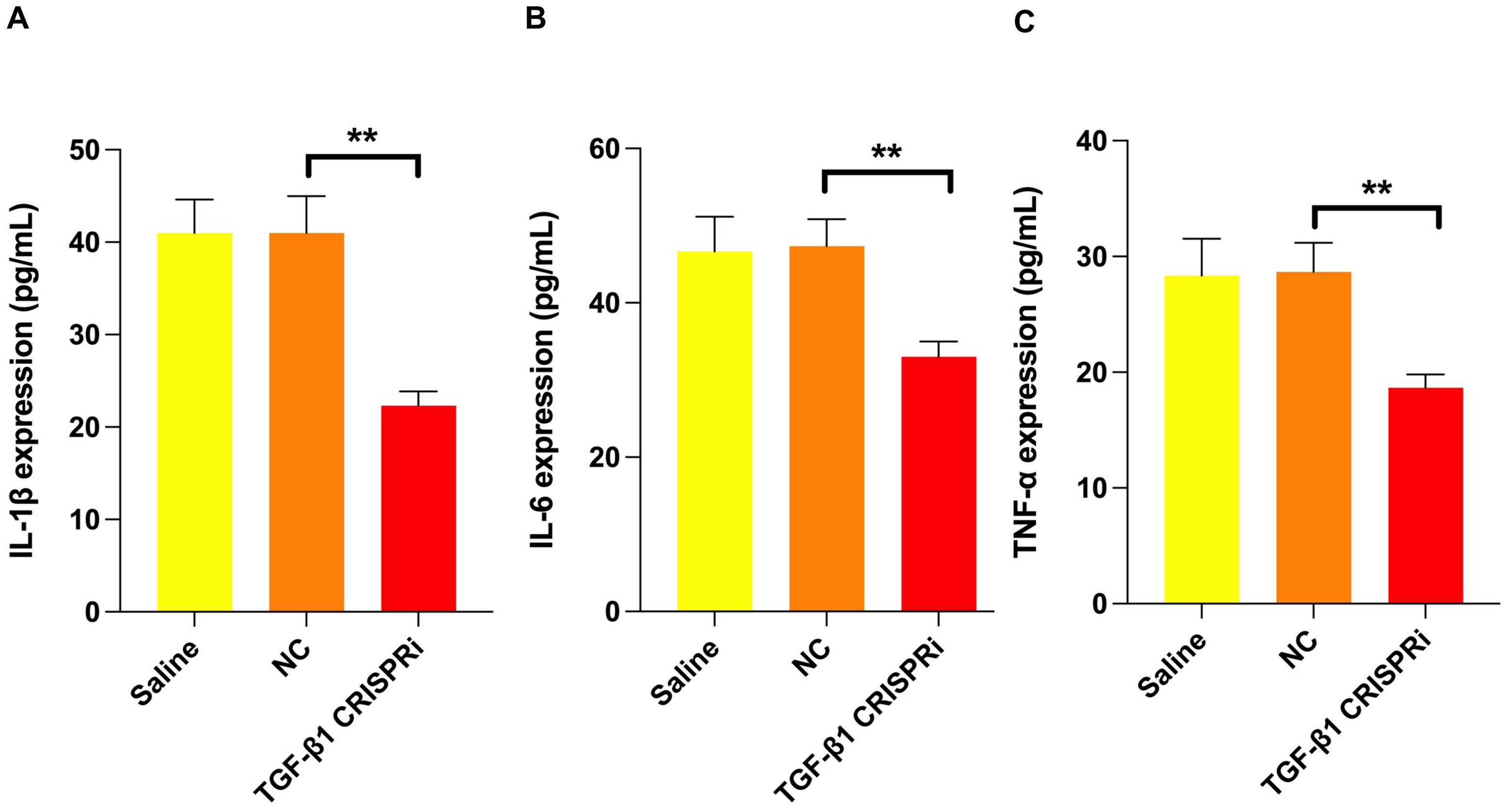

Figure S10. ELISA assay demonstrated the protein levels of IL-1β (A), IL-6 (B), and TNF-α (D) from midbrain of mice. Data were presented as means ± sd. The experiments were carried out three times (n=3). One-way ANOVA followed by Tukey's multiple comparison test in (A, B, C). The difference in folds is statistically significant. $^{**}P < 0.01$.

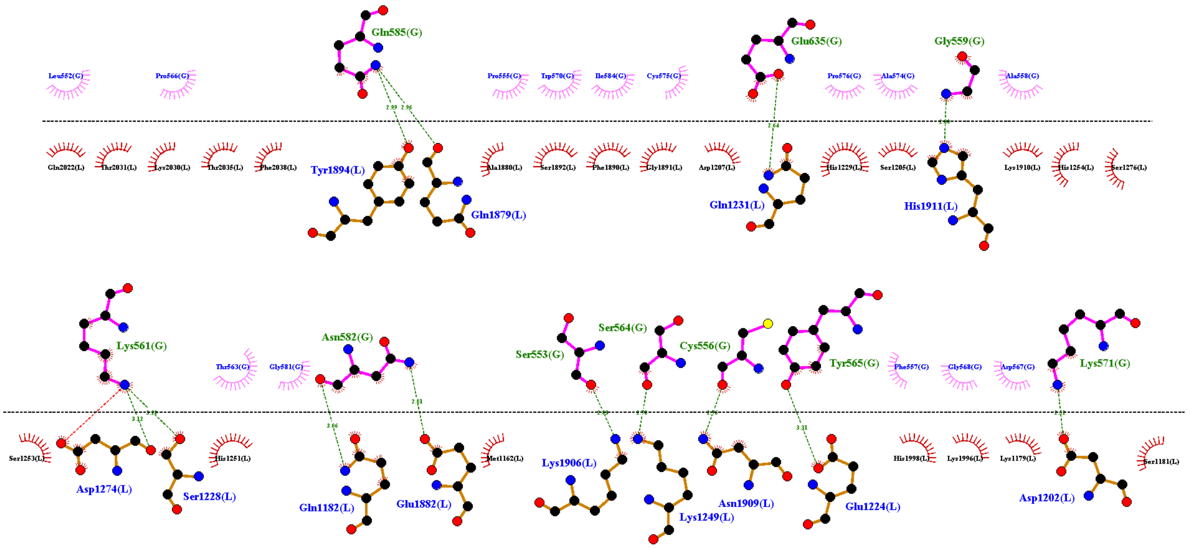

Figure S11. Two-dimensional interaction map of LRRK2 and THBS1. The "T chain" refers to THBS1, and the "L chain" refers to LRRK2. The rod-like structures represent the interacting amino acid residues. In the red-black diagram, the red dashed lines represent salt bridge interactions, while the green dashed lines represent hydrogen bond interactions. For example, a salt bridge interaction occurs between the negatively charged ASP1274 in LRRK2 and the positively charged LYS561 in THBS1 protein.

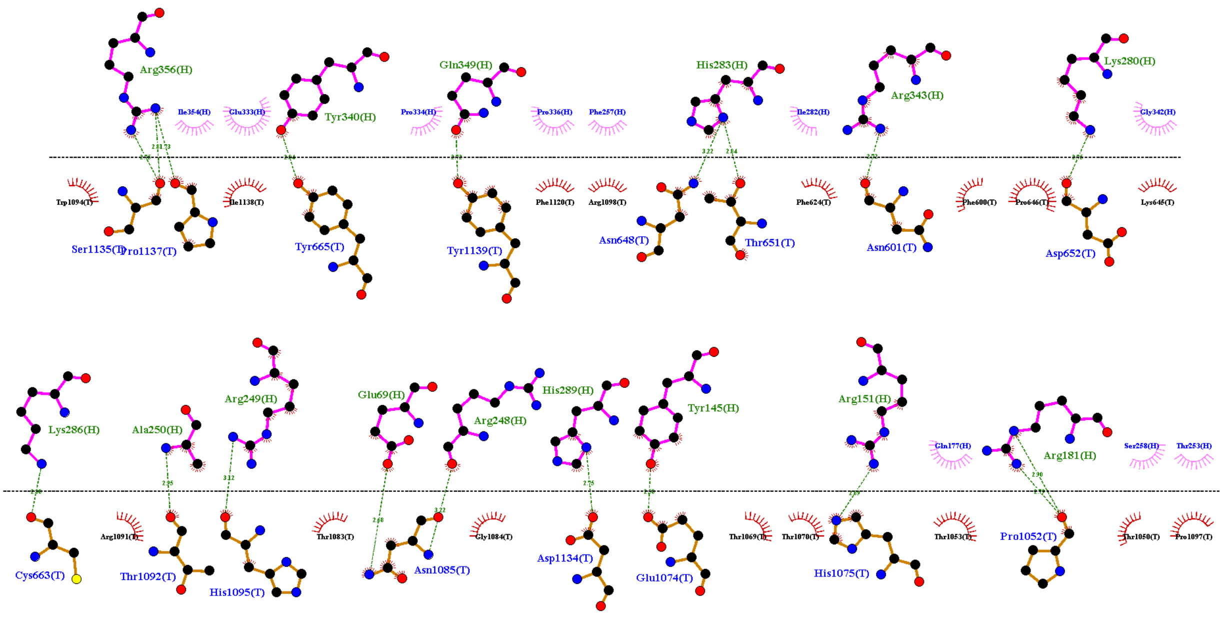

Figure S12. Two-dimensional interaction map of THBS1 and TGF-β1. The "T chain" represents THBS1, and the "H chain" represents TGF-β1. The rod-like structures represent the interacting amino acid residues. In the red-black diagram, the red dashed lines represent salt bridge interactions, while the green dashed lines represent hydrogen bond interactions.
