# Supplementary material for: LRRK2 Gly2019Ser Mutation Promotes ER Stress via Interacting with THBS1/TGF‐β1 in Parkinson's Disease
Source: Adv Sci (Weinh). 2023 Sep 6;10(30):2303711. doi: 10.1002/advs.202303711 (PMC10602550; doi:10.1002/advs.202303711)
Supplement: Supplementary file 2 — Supplemental Table 1 [file ADVS-10-2303711-s001.pdf]

ADVANCED SCIENCE
Open Access

Supporting Information

for *Adv. Sci.*, DOI 10.1002/advs.202303711

LRRK2 Gly2019Ser Mutation Promotes ER Stress via Interacting with THBS1/TGF-β1 in Parkinson's Disease

Longping Yao, Fengfei Lu, Sumeyye Koc, Zijian Zheng, Baoyan Wang, Shizhong Zhang*, Thomas Skutella* and Guohui Lu**

Table S1. Top 20 clusters with their representative enriched terms (one per cluster). The number of genes in the user-provided lists that belong to the supplied ontology term is called "count." "%" is the percentage of all of the user-provided genes that are found in the given ontology term (only input genes with at least one ontology term annotation are included in the calculation). "Log10(*P*)" is the *P*-value in log base 10. "Log10(q)" is the multi-test adjusted p-value in log base 10.

GO	Category	Description	Count	%	Log10(P)	Log10(q)
**GO:0001568**	GO Biological Processes	blood vessel development	76	11.55	-27.86	-23.5
**GO:0048729**	GO Biological Processes	tissue morphogenesis	65	9.88	-25.35	-21.29
**GO:0007507**	GO Biological Processes	heart development	63	9.57	-24.78	-20.89
**R-HSA-9006934**	Reactome Gene Sets	Signalling by Receptor Tyrosine Kinases	59	8.97	-24.53	-20.77
**GO:0032989**	GO Biological Processes	cellular component morphogenesis	70	10.64	-24.24	-20.58
**GO:0070848**	GO Biological Processes	response to growth factor	68	10.33	-23.57	-19.99
**R-HSA-1474244**	Reactome Gene Sets	Extracellular matrix organization	44	6.69	-22.82	-19.31
**WP5087**	WikiPathways	Malignant pleural mesothelioma	50	7.6	-21.38	-17.97
**GO:0007169**	GO Biological Processes	transmembrane receptor protein tyrosine kinase signalling pathway	60	9.12	-21.05	-17.82
**GO:0009611**	GO Biological Processes	response to wounding	56	8.51	-20.97	-17.81

**WP5094**	WikiPathways	Orexin receptor pathway	31	4.71	-20.35	-17.26
**GO:0061061**	GO Biological Processes	muscle structure development	59	8.97	-20.07	-17.01
**GO:0060322**	GO Biological Processes	head development	66	10.03	-19.88	-16.84
**GO:0008285**	GO Biological Processes	negative regulation of cell population proliferation	65	9.88	-19.72	-16.71
**GO:0048732**	GO Biological Processes	gland development	48	7.29	-19.45	-16.47
**GO:0030155**	GO Biological Processes	regulation of cell adhesion	63	9.57	-19.24	-16.29
**GO:0007423**	GO Biological Processes	sensory organ development	53	8.05	-18.71	-15.81
**GO:0010942**	GO Biological Processes	positive regulation of cell death	56	8.51	-18.66	-15.77
**GO:0034330**	GO Biological Processes	cell junction organization	59	8.97	-18.25	-15.41
**GO:0060485**	GO Biological Processes	mesenchyme development	37	5.62	-17.15	-14.45
