# Supplementary material for: LRRK2 Gly2019Ser Mutation Promotes ER Stress via Interacting with THBS1/TGF‐β1 in Parkinson's Disease
Source: Adv Sci (Weinh). 2023 Sep 6;10(30):2303711. doi: 10.1002/advs.202303711 (PMC10602550; doi:10.1002/advs.202303711)
Supplement: Supplementary file 3 — Supplemental Table 2 [file ADVS-10-2303711-s002.pdf]

ADVANCED SCIENCE
Open Access

Supporting Information

for *Adv. Sci.*, DOI 10.1002/advs.202303711

LRRK2 Gly2019Ser Mutation Promotes ER Stress via Interacting with THBS1/TGF-β1 in Parkinson's Disease

Longping Yao, Fengfei Lu, Sumeyye Koc, Zijian Zheng, Baoyan Wang, Shizhong Zhang*, Thomas Skutella* and Guohui Lu**

Table S2. Summary of enrichment analysis in Transcription Factor Targets

GO	Description	Count	%	Log10(P)	Log10(q)
**M572**	TGCCAAR NF1 Q6	63	9.6	-20	-17
**M4764**	TGTTTGY HNF3 Q6	60	9.1	-17	-15
**M17712**	WGTTNNNNNAAA UNKNOWN	50	7.6	-17	-14
**M6517**	AAAYWAACM HFH4 01	34	5.2	-16	-13
**M17420**	WGGAATGY TEF1 Q6	37	5.6	-13	-11
**M14357**	TATA C	31	4.7	-13	-10
**M14351**	AFP1 Q6	30	4.6	-13	-10
**M8720**	STAT5A 03	30	4.6	-12	-10
**M16291**	YNGTTNNNATT UNKNOWN	35	5.3	-12	-9.9
**M1328**	WTGAAAT UNKNOWN	45	6.8	-12	-9.3
**M16417**	FOXO4 02	28	4.3	-11	-9
**M822**	YTATTTTNR MEF2 02	48	7.3	-11	-8.9
**M946**	GGATTA PITX2 Q2	43	6.5	-11	-8.9
**M9612**	OCT1 01	28	4.3	-11	-8.7
**M11838**	FOXD3 01	24	3.6	-11	-8.5
**M9329**	SRY 02	27	4.1	-11	-8.5
**M4815**	WTTGKCTG UNKNOWN	39	5.9	-11	-8.4
**M399**	SMTTTTGT UNKNOWN	34	5.2	-11	-8.4
**M1031**	FREAC2 01	27	4.1	-10	-8.3
**M19327**	CDC5 01	27	4.1	-10	-8.2
